# COVID-19 infection and mortality among non-pregnant indigenous adults in Mexico 2020-2022: Impact of marginalisation

**DOI:** 10.7189/jogh.13.06030

**Published:** 2023-07-28

**Authors:** Bert B Little, Shaminul Shakib, Maria E Pena Reyes, Seyed Karimi, Giang T Vu, Natalie Dupré, W Paul McKinney, Riten Mitra

**Affiliations:** 1 School of Public Health and Information Sciences, University of Louisville, Kentucky, USA; 2 Escuela de Nacional Antroplogia e Historia and Instituto de Nacional Antroplogia e Historia Mexico City, Mexico; 3 School of Global Health Management and Informatics, University of Central Florida, Orlando, Florida, USA

## Abstract

**Background:**

Indigenous individuals have higher rates of mortality and poverty in Mexico and more than half are marginalised, and COVID-19 pandemic aggravated the existing burden of health disparities. We aimed to analyse the effects of being indigenous and marginalised on coronavirus (COVID-19) infection fatality in Mexico.

**Methods:**

We identified 3 424 690 non-pregnant, COVID-19 positive adults ≥19 years in the Mexico national COVID-19 database with known date of symptom. We used demographic information, indigenous status, marginalisation status, and co-morbidities in binary logistic regression to predict mortality, adjusting for covariates, including hospitalisation, admission to the intensive care unit (ICU), and mechanical ventilation use. We also assessed the interaction between indigenous status and marginalisation.

**Results:**

Marginalisation was much higher among indigenous (53.7%) compared to non-indigenous individuals (4.8%). COVID-19 fatalities were approximately 20 years older (64.4 and 63.0 years) than survivors (44.7 and 41.2 years) among indigenous vs non-indigenous individuals, respectively. The unadjusted risk of COVID-19 fatality among indigenous individuals was nearly two-fold (odds ratio (OR) = 1.92)) compared to non-indigenous individuals (OR = 1.05). COVID-19 fatality was higher among highly marginalised individuals (upper quartile) (OR = 1.51; 95% confidence interval (CI) = 1.49-1.54). Marginalised indigenous individuals had a significantly lower likelihood of ICU admission compared to non-indigenous non-marginalised individuals. The likelihood of mechanical ventilation for indigenous individuals was 4% higher compared to non-indigenous individuals. Indigenous marginalised individuals had a significantly lower probability of mechanical ventilation compared to non-indigenous non-marginalised individuals. COVID-19 comorbidity risks of fatality significantly differed between the two groups in the Cox survival analysis. In the fully adjusted model, indigenous individuals were 4% more likely to die from COVID-19 compared to non-indigenous.

**Conclusions:**

Indigenous, marginalised individuals with COVID-19 had higher risk of hospitalisation and ICU admission than non-indigenous patients. Marginalised, indigenous individuals were less likely to receive mechanical ventilation compared to non-indigenous, but had a higher risk of COVID-19. Indigenous individuals had a 4% higher COVID-19 mortality risk COVID-19 compared to non-indigenous individuals. Improved community medical care and augmented health services in rural hospitals could mitigate barriers to health care access in indigenous, marginalised populations.

Indigenous people are often marginalised and have high rates of chronic diseases [1,2], some of which (including type two diabetes (T2DM), chronic kidney disease (CKD), cardiovascular disease (CVD), and chronic obstructive pulmonary disease (COPD)) hinder their health status [3]. Their communities are frequently geographically isolated, separated due to low socioeconomic status, and usually have limited access to health care. Likewise, they tend to suffer more from infectious diseases and chronic diseases [4,5]. Indigenous populations in Mexico are of Amerindian ancestry, and such populations in the USA have markedly higher rates of coronavirus (COVID-19) infection and mortality than non-Amerindian groups [6].

During the COVID-19 pandemic in Mexico, indigenous communities had limited medical care access compared to non-indigenous people [7], with a COVID-19 mortality rate of 9.7% (97 per 1000 population) [8]. The weekly crude COVID-19 fatality rate per 1000 persons was 64.8% higher among indigenous individuals compared to non-indigenous individuals in Mexico [9]. Reasons for poorer health status among indigenous peoples include sub-optimal nutrition, no or limited access to acute health care, longer distances from indigenous communities to rural hospitals, lower tiers of care in rural hospitals, and longer distances from rural hospitals to urban centers with intensive care units (ICU) [5,10]. In the USA, COVID-19-related excess mortality in indigenous communities was drastically higher than in non-indigenous groups. Reasons for poorer health status among indigenous peoples include sub-optimal nutrition, no or limited access to acute health care, long distances from Indigenous communities to rural hospitals, lower tier of care in rural hospital, and long distances from rural hospitals to urban centres with intensive care unit [11]. COVID-19 mortality in indigenous communities in Mexico has not been studied after adjusting for comorbidities, but it likely follows similar trends of higher COVID-19 mortality risk individuals >60 years old as in the USA [12]. Comorbidities associated with higher COVID-19 mortality rates include pneumonia, CVD, T2DM, CKD, COPD, asthma, and, in some cases, smoking tobacco [13-16]. In contrast, limited evidence suggests smoking tobacco can be protective against COVID-19 hospitalisation [17], but the weight of the evidence points to smoking as a risk factor [18]. Survival of indigenous individuals following COVID-19 infection was 13% lower compared to non-indigenous individuals in Mexico [19], but the estimate was not adjusted for potential confounders. The effect of marginalisation on indigenous and marginalised communities for COVID-19 outcomes has not been studied.

Indigenous individuals are reported to have a higher crude fatality rate than non-indigenous patients (29.97 vs. 18.18 per 1000 person-weeks, respectively) [9,20] and a higher observed fatality rate compared to non-indigenous individuals (16.5% vs 11.1%, respectively) [19]. Another study reported an even greater disparity in COVID-19 related fatalities among indigenous (14.5%) vs non-indigenous individuals (4.9%) [21]

Rural, marginalised indigenous communities in Mexico usually only have access to medical clinics with small pharmacies staffed by one or more nurses and a physician at least one day a week; the physician is frequently a recently graduated medical school student doing regular required community service residency. Mexico has a national health care system supported by Mexican Social Security Institute (IMSS). Even in remote communities, they have a desktop computer and a database system for patient records. From our field experience on other projects, we noted that, for patients who missed medical clinic appointments, a nurse would either conduct an assessment during a household visit, or, if necessary, physically transport them to the clinic. Transportation is often a barrier to rural clinic referrals to hospitals.

We aimed to evaluate the natural history of COVID-19 starting at diagnosis and following through hospitalisation, ICU admission, ventilation use, and death among marginalised and non-marginalised indigenous non-pregnant individuals aged ≥19 years compared to non-indigenous adults in Mexico.

## METHODS

This observational study was based on a secondary analysis of data collected between 1 March 2020 and 28 February 2022 from 10 487 563 individuals. These data are openly available at the public national COVID-19 database from the Ministry of Health, Gobierno México, Mexico City D.F. [22], and were used in several studies due to their reliability and accessibility for ease of replication.

**Figure 1:**
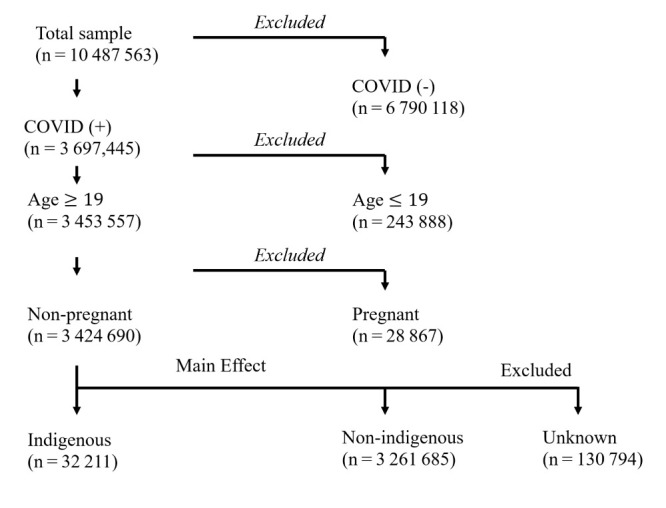
Sample inclusion/exclusion criteria.

We included non-pregnant adults aged ≥19 years old who were confirmed to be COVID-19 positive through a laboratory-tested sample or antigen test in Mexico’s Clinical Epidemiology COVID-19 database (**Figure 1**). We only included individuals whose status was confirmed as indigenous (n = 32 211) (by self-report and language spoken) or non-indigenous (n = 3 261 685), excluding individuals whose status was unknown (n = 130 794). Missing data were deleted listwise (Table S6 in the **Online Supplementary Document**).

We recoded the data using the database dictionary [22] and structured it for analysis. We used logistic regression analysis to analyse hospital admissions, ICU admissions, and mechanical ventilation (see Table S2 in the **Online Supplementary Document** for Raw unadjusted odds ratios (uORs)) and evaluated its fit using the Hosmer and Lemeshow goodness of fit statistic. We calculated sensitivity and specificity on the whole sample. We drew a random sample (n = 25 000) for each analysis because the test is biased at larger sample sizes [23]. We used Cox regression to analyse fatalities from March 2020 to February 2022, and the χ^2^ goodness of fit test to evaluate the goodness of fit of the models.

Age, days from symptom onset to hospitalisation, and days to death from symptom onset were continuous variables. We recoded age into <60 years and ≥60 based on best risk splits observed in the Mexico data, sex as 0 = female, and the indicator variables in the clinical epidemiology data set to binary values (0 and 1), where 1 = yes, and included pneumonia, diabetes (type unspecified), COPD, asthma, hypertension, CVD, obesity, CKD, immunosuppression, and smoking. Indigenous status and speaking an indigenous language was self-reported and missing for 3.95% of the sample. We calculated number of days to hospitalisation by subtracting the date of admission from the date of onset of symptoms. For the Cox regression, we calculated days to death as the difference between the onset of symptoms and the date of death.

The Mexican government’s marginalisation index is based on the National Council of Population’s (CONAPO) Index of Marginalization [24]. It is a summary measure at the *municipio* level where higher values indicate higher marginalisation. It includes total census population and percentages of illiteracy in population, those aged ≥15 years without basic education (reading), populations living in communities less than 5000 people, and households without a drainage system, households without electricity, households without running water in the house, overcrowded households, households with dirt floors, and households with two or more minimum wage salaries. This produces a marginalisation index classed as a range between I (very low) and VI (very high), with higher values indicating greater marginalisation. We obtained the raw numeric and recoded indices. We merged the index at the *municipio* (a unit of local government including several villages or barrios) level for each individual *municipio* of residence. The index also quantifies access to education, inadequate housing, insufficient monetary income, geographic isolation by distance, and residence in sparsely populated locations.

We transformed marginalisation from a continuous variable to a binary one (0, 1) using the fourth quartile of marginalisation to indicate the highest 25% of *municipio* marginalisation found in the Mexico Index = 1, thus flagging those at the extreme as marginalised. We optimised the coding at the fourth interquartile. We previously used the marginalisation index and found it was associated with significantly reduced child growth at higher levels of marginalisation [25].

We determined the odds of being admitted to a hospital, admitted to an ICU, and being placed on a mechanical ventilator by logistic regression. We conducted a Cox regression analysis of death (days to death from symptoms to death = time), controlling for all covariates including ICU admission, mechanical ventilation use, and days to hospital admission from the onset of symptoms. We conducted analyses for the entire sample and separately for indigenous vs non-indigenous status, allowing for comparison of the effects of comorbidities between indigenous and non-indigenous groups to test factors that may differ between the two groups in hospital and ICU admission, mechanical ventilator use, and fatality through their 95% confidence intervals (CIs). The adjusted odds ratio (aOR) indicates that covariates in the model are held constant (see Tables S1 to S3 in the **Online Supplementary Document** for uORs with 95% CIs). We calculated an interaction term using indigenous status and marginalisation. We repeated all regression, controlling for the same covariates with the addition of the interaction term variable. We only presented the interaction results in Table S5 in the **Online Supplementary Document** for brevity. We conducted all analyses using SPSS v28 (IBM SPSS, Chicago, IL, USA) and SAS v9.4 (SAS Institute, Cary, NC).

## RESULTS

### Descriptive statistics

Approximately 53.7% of indigenous individuals and 4.8% of non-indigenous individuals were from marginalised communities. Mean age was different by about 20 years between COVID-19 survivors and fatalities (**Table 1**). The unadjusted odds ratio (uOR) of being infected with COVID-19 indicated that the odds increased to 1.05 among indigenous individuals compared to non-indigenous individuals. The uOR of dying from COVID-19 was almost two-fold among indigenous individuals compared to non-indigenous individuals. The uOR of death among the high (upper quartile) marginalisation group was significantly higher compared to the non-marginalised group (**Table 1**).

**Table 1:** Descriptive statistics of COVID-19 among adults in Mexico (n = 10 487 563 total and 3 424 690 analysed): January 2020 to February 2022*

Variable	Indigenous			Non-indigenous				
	**Total (n)**	**Positive, n (%)**	**Negative, n (%)**	**Total (n)**	**Positive, n (%)**	**Negative, n (%)**	**OR (95% CI)†**	***P***
COVID-19 infection‡	41 824	15 287 (36.6)	26 537 (63.4)	4 658 093	1 649 096 (35.4)	3 008 997 (64.6)	1.05 (1.03-1.07)	
		**Alive, n (%)**	**Dead, n (%)**		**Alive, n (%)**	**Dead, n (%)**		
COVID-19 mortality	32 211	28 234 (87.7)	3977 (12.3)	3 261 685	3 039 284 (93.2)	222 401 (6.8)	1.92 (1.86-1.99)	
Marginalisation		15 118 (87.5)	2163 (12.5)		140 498 (90.6)	14 593 (9.4)	23.2 (22.7-23.7)	
Age, mean (SD)		44.65 (16.11)	64.35 (14.00)		41.18 **(**14.62)	63.14 (14.35)		<0.001 (alive group), <0.006§ (dead group)§¶
Age, median (IQR)		43.00, 32.00-55.00	65.00, 56.00-74.00		39.00, 29.00-51.00	64.00, 54.00-73.00		
Male		14 436 (85.5)	2448 (14.5)		1 467 359 (91.5)	136 164 (8.5)	1.14 (1.11-1.16)	
Female		13 798 (90)	1529 (10)		1 571 925 (94.8)	86 237 (5.2)		
Diabetes		3893 (72.3)	1490 (27.7)		280 644 (77.3)	82 191 (22.7)	1.60 (1.56-1.65)	
COPD		467 (64.3)	259 (35.7)		19 015 (66.3)	9659 (33.7)	2.60 (2.41-2.80)	
Asthma		564 (85.1)	99 (14.9)		59 442 (93.7)	3964 (6.3)	1.06 (0.98-1.15)	
Pneumonia		3202 (50.5)	3142 (49.5)		185 824 (53.7)	160 071 (46.3)	2.07 (2.01-2.13)	
Hypertension		4302 (73.3)	1569 (26.7)		380 832 (79.)	99 425 (20.7)	1.29 (1.25-1.33)	
Cardiovascular		349 (67.1)	171 (32.9)		28 308 (72)	10 998 (28)	1.34 (1.23-1.48)	
Obesity		4320 (82.3)	926 (17.7)		354 006 (88)	48 467 (12)	1.38 (1.34-1.42)	
CKD		300 (61.7)	186 (38.3)		23 794 (61)	15 214 (39)	1.27 (1.16-1.39)	
Immunosuppressed		178 (71.8)	70 (28.2)		15 623 (76.8)	4718 (23.2)	1.24 (1.09-1.40)	
Smoking		1379 (86.5)	216 (13.5)		192 890 (92.3)	16 171 (7.7)	0.76 (0.72-0.80)	

OR – odds ratio, CI – confidence interval, SD – standard deviation, COPD – chronic obstructive pulmonary disease, CKD – chronic kidney disease, IQR – interquartile range

*Odds of death among highly marginalised group is significant (OR = 1.51; 95% CI = 1.49-1.54).

†OR compares indigenous (coded as 1) vs non-indigenous (coded as 0).

‡COVID-19 infection include cases who are non-pregnant and aged ≥19.

§Independent group *t*-test compares age between indigenous and non-indigenous for alive.

¶Independent group *t*-test compares age between indigenous and non-indigenous for dead.

### Hospitalisation

**Figure 2:**
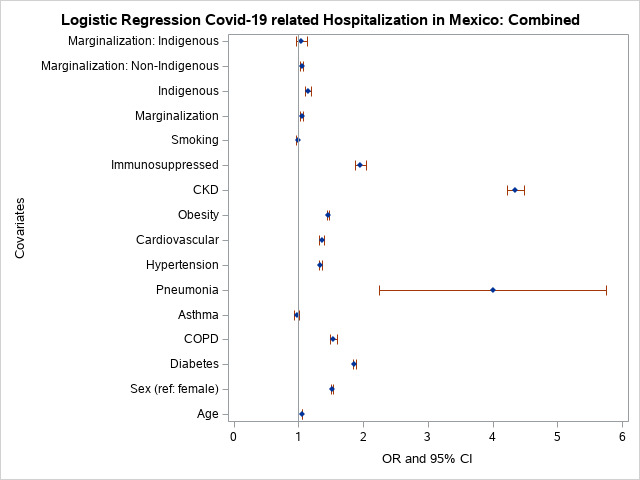
Odds of hospitalisation in Mexico.

We used logistic regression to analyse the effects of indigenous status and marginalisation (>75^th^ percentile) on hospitalisation, adjusting for potential confounders (age (>60 years), sex, diabetes, COPD, asthma, pneumonia, hypertension, CVD, obesity, CKD, immunosuppression, and tobacco use). For the full sample, marginalisation had an OR of 1.05 for hospitalisation. The effect of marginalisation on hospitalisation, adjusted for covariates, indicated that non-indigenous individuals were more likely to be hospitalised compared to indigenous individuals (**Figure 2**) (Table S1 in the **Online Supplementary Document**). Indigenous individuals who lived in marginalised areas were not significantly more likely to get hospitalised than non-indigenous patients. Older male patients with diabetes, COPD, pneumonia, hypertension, CVD, obesity, CKD, immunosuppression, and those who were indigenous had higher odds of being hospitalised (Table S1 in the **Online Supplementary Document**).

### ICU admission

In the combined sample, marginalised individuals had significantly increased odds of ICU admission. Marginalisation and ICU admission showed that non-indigenous individuals were significantly more likely to be admitted to ICUs (Table S2 in the **Online Supplementary Document**). ICU admissions were higher among those who were ≥60 years, males, had diabetes, pneumonia, CVD, obesity, or immunosuppression, and were indigenous (Table S2 in the **Online Supplementary Document**).

COPD, CKD, and smoking significantly decreased the odds of ICU admission. Non-indigenous individuals from high marginalisation areas had higher odds of ICU admission compared to indigenous individuals. The combined model that included the interaction term between indigenous and marginalisation showed that indigenous marginalised individuals were significantly less likely to be admitted to the ICU (Table S5 in the **Online Supplementary Document**) compared to those who were neither marginalised nor indigenous.

### Mechanical ventilation

In the combined sample, odds of mechanical ventilation use were higher in those ≥60 years. Hazards of fatality were also higher among males, and those with diabetes, asthma, pneumonia, hypertension, obesity, and immunosuppression (Table S3 in the **Online Supplementary Document**). COPD and CKD decreased the odds of being placed on mechanical ventilation. In the combined sample, mechanical ventilation use odds were not significantly different between indigenous and non-indigenous individuals. In contrast, living in a marginalised *municipio* significantly decreased the odds of being placed on a mechanical ventilator, regardless of indigenous status, and indigenous individuals in marginalised areas were significantly less likely to receive mechanical ventilation than non-indigenous individuals in marginalised areas (Table S3 in the **Online Supplementary Document**), which is supported by the interaction term between indigenous and marginalisation in this group. In the interaction model, indigenous marginalised individuals were significantly less likely to receive mechanical ventilation compared to those who were not marginalised and not indigenous (Table S5 in the **Online Supplementary Document**).

### Cox regression analysis of death

**Figure 3:**
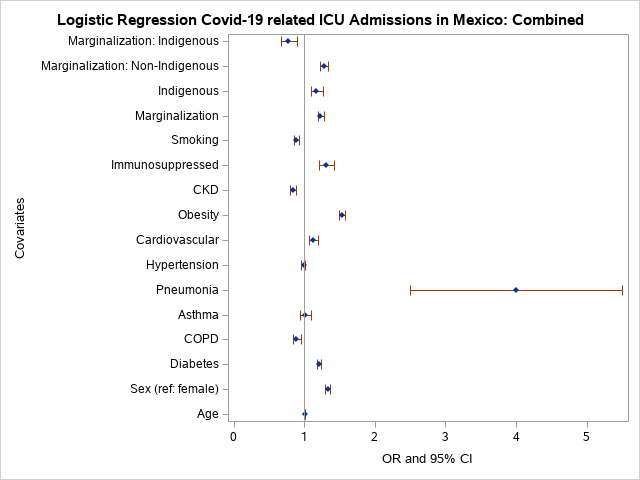
Odds of ICU admission in Mexico.

Survival analysis of the combined sample indicated that COVID-19-related fatality indicated males were at significantly higher risk than females to die from COVID-19-related complications. Hazards for fatality were higher among those of older age (≥60 years), with diabetes, pneumonia, hypertension, obesity, CKD, immunosuppression, patients on mechanical ventilation, and among the indigenous (**Figure 3**, Table S4 in the **Online Supplementary Document**). Of these comorbidities, pneumonia fatality risk was significantly higher among indigenous individuals compared to non-indigenous individuals. Fatality risk was higher among obese indigenous compared to obese non-indigenous individuals. Hazards of fatality for CKD and mechanical ventilation use were not significantly different between non-indigenous and indigenous individuals. Similarly, COVID-19-related fatality risk was not significantly higher among indigenous individuals who had asthma. Among non-indigenous individuals, asthma decreased the likelihood of fatality for non-indigenous individuals. In the interaction model, the risk of fatality of marginalised indigenous individuals was not higher (**Figure 3**, Table S5 in the **Online Supplementary Document**).

## DISCUSSION

We found that indigenous and marginalised patients who were positive for COVID-19 had a higher risk of hospitalisation and ICU admission than non-indigenous patients, but had a lower probability of being placed on mechanical ventilation. Adjusted fatality risk among COVID-19 positive indigenous individuals was 4% higher than among non-indigenous individuals after adjusting for all covariates. In the fully adjusted model, the marginalisation effect was not significant. These results suggest the need for improved community medical care capabilities and augmented health services in the rural hospitals. Improved resources at the community level (testing, pneumonia treatment) and at the regional rural hospitals (i.e. ICU, ventilator availability) may mitigate barriers to health care availability and access for indigenous and marginalised populations.

Approximately 15.1% of Mexico’s population are indigenous individuals (n = 16 933 283) who live in 64 172 communities [26], most of which are marginalised to some extent. In this study, we used the most extreme definition for marginalisation – the top 25% (fourth quartile). Eight of the 32 states and the Federal District are home to 85% of the indigenous population in Mexico: Chiapas, Hidalgo, San Luis Potosi, Oaxaca, Yucatan, Veracruz, Durango, and Guerrero. The 10 most populous indigenous groups include Nahuatl (24%), Maya (13.7%), Mixtec (6.8%), Zapotec (6.8%), Tseltal (5.7%), Paipai (5.5%), Otomi (5.5%), Tsotsil (5.1%), Totonaco (3.6%), and Mazahua (3.0%). The COVID-19 indigenous sample population studied represented 0.98% of total cases in the national registry, meaning that indigenous people are 15-fold under-represented in the sample, indicating a large bias in under sampling. One possible explanation is that many indigenous individuals are often extremely isolated and died at home from COVID-19-related causes without entering the health care system and being captured in the COVID-19 surveillance system. Gaps in COVID-19 reporting in Mexico have been previously observed [20]. According to one study, indigenous individuals in Mexico have a 13% higher risk of death from COVID-19 compared to non-indigenous individuals [19]. We found that the unadjusted odds of COVID-19-related death was 1.92 times higher among indigenous individuals compared to non-indigenous individuals. The fully adjusted odds of COVID-19-related death was reduced to 4% between indigenous individuals and non-indigenous individuals (**Figure 3**) (Table S4 in the **Online Supplementary Document**). Further, being from a highly marginalised area, regardless of indigenous status, increased COVID-19-related probability of death by 1.51-fold. All the risk factors analysed were modifiable except for age, sex, and indigenous status.

Our analysis indicated that indigenous individuals had an increased odds of being hospitalised, and individuals from marginalised groups had a small (5%) significantly higher risk of hospitalisation (aOR = 1.05; 95% CI = 1.03-1.07) compared to those from non-marginalised groups (**Figure 2**) (Table S1 in the **Online Supplementary Document**). Travelling distance between indigenous communities and rural hospitals are lengthy and limit hospital access compared to non-indigenous communities. The marginalisation index includes distance to medical and urban centres, a major contributor to the index. In the combined analysis of hospitalisation, asthma (aOR = 0.98; 95% CI = 0.94-1.01) was not significant and smoking (aOR = 0.99; 95% CI = 0.97-1.00) borderline lowered probability of hospital admission. Our findings are in line with a study on COVID-19 infection and hospitalisation in Mexico [27]. However, asthma-related risk of hospitalisation for indigenous individuals was 1.3-fold higher, while it significantly lowered the probability of hospital admission for non-indigenous individuals. It is possible that asthma is better controlled in non-marginalised areas, perhaps with over-the-counter inhaled corticosteroids such as dexamethasone (i.e. glucocorticoid), which was very effective in treating COVID-19 pneumonia [28].

CKD also resulted in different hospitalisation rates among indigenous and non-indigenous individuals. Non-indigenous individuals with CKD were more than four-fold more likely to be hospitalised (aOR = 4.39; 95% CI = 4.52-4.52), and indigenous individuals with CKD were nearly two times more likely than those without CKD. The high odds of hospitalisation for COVID-19 patients with CKD aligns with prior research [27].

For ICU admission, indigenous and marginalised individuals were 1.17 and 1.23 more likely to be hospitalised, respectively, but the difference in these odds was not statistically significant (**Figure 3** and Table S2 in the **Online Supplementary Document**). Indigenous marginalised individuals had a 1.29-fold lower likelihood of admission to ICU units, but non-indigenous marginalised individuals had a 1.28 times increased likelihood. Analysis of the combined sample indicated that COPD, CKD, and smoking lowered the likelihood of being admitted to the ICU. The likelihood of ICU admission was increased by 43.10-fold among indigenous individuals with pneumonia, significantly higher compared to non-indigenous individuals whose odds were 28.78-fold higher than for non-indigenous individuals without pneumonia.

**Figure 4:**
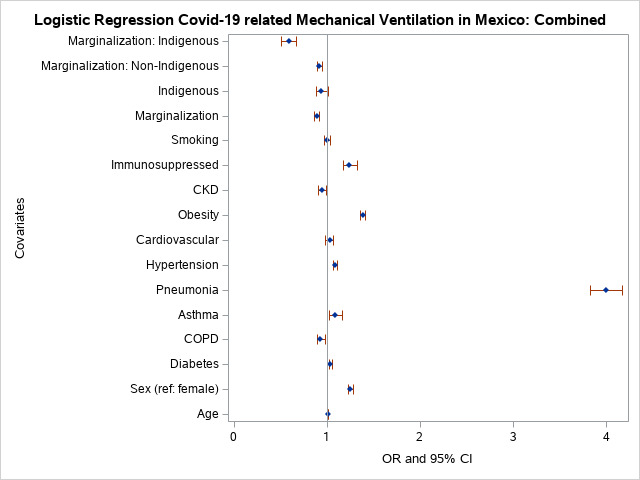
Odds of mechanical ventilaton in Mexico.

Furthermore, individuals from marginalised groups were significantly less likely to be placed on mechanical ventilation compared to non-indigenous individuals. However, in the model with the interaction term (marginalisation × indigenous), indigenous individuals from marginalised areas were significantly (1.56 times) less likely to receive mechanical ventilation compared to non-indigenous and marginalised individuals (Table S5 in the **Online Supplementary Document**). COPD and CKD significantly decreased the likelihood of patients being placed on mechanical ventilator (**Figure 4**) (Table S3 in the **Online Supplementary Document**).

### Diabetes

Diabetes is disproportionately distributed between indigenous and non-indigenous people. Cross-sectional studies on 12 unique indigenous groups in Mexico indicated that T2DM prevalence ranged from 2% to 6% [29,30]. Diabetes is associated with an impaired immune response, increasing incidence and severity of COVID-19 infection [31]. Thus, indigenous individuals living in poor health and nutritional conditions with diabetes are at a high risk of COVID-19 infection and fatality. We found that indigenous individuals were significantly (four-fold) more likely to die from COVID-19 compared to non-indigenous individuals in the fully adjusted combined model (Table S4 in the **Online Supplementary Document**). Diabetes was associated with a 1.12-fold increased risk of COVID-19 related fatality. COVID-19 fatality hazard ratio (HR) was not statistically different between groups. The HR of fatality was 1.18 among the indigenous and 1.12 among the non-indigenous group, which is not significantly different from the non-indigenous hazard for death. This finding is in contrast with other studies, which observed a higher rate of diabetes and complications among indigenous people [32].

### COPD

Tobacco smoking is the most common COPD risk factor [33], with the highest COPD prevalence among Amerindian adults [34] and those who reside in poor, rural, and marginalised areas [35]. Individuals with COPD and COVID-19 have an increased risk of severe pneumonia and poor outcomes [36]. Severe COVID-19 risk is approximately four times higher among individuals with COPD compared to those without COPD [37]. In our study, COPD was not associated with an increased risk of COVID-19 mortality. We hypothesise that COPD contributed to COVID-19 pneumonia, which was a significant morality risk factor. COPD became statistically significant (*P* < 0.001) when we excluded pneumonia from the model. Therefore, we reasoned that the variance accounted for by pneumonia included COPD effects, which is biologically plausible.

### Asthma

**Figure 5:**
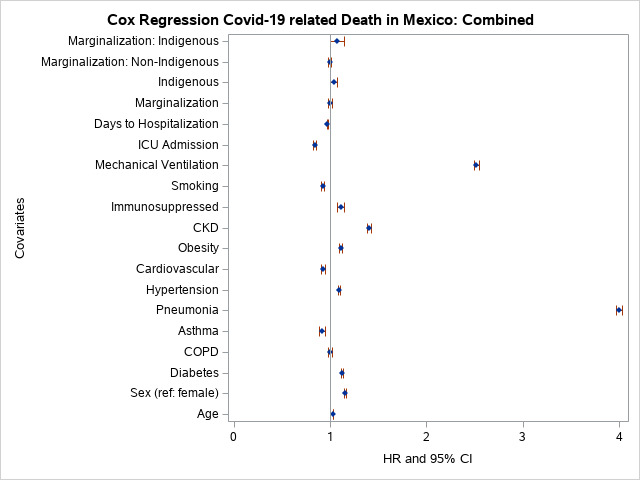
Hazard ratios of mortality in Mexico.

The relationship between COVID-19 and asthma is not consistent across studies. Asthma was not a significant risk factor for COVID-19 severity after adjusting for body mass index (BMI) and age in one study [38]. In our model, asthma lowered the probability 1.09-fold of COVID-19 fatality, possibly related to glucocorticoid asthma treatments available over the counter in Mexico. Other analyses of COVID-19 mortality in Mexico have reported reduced risk of hospitalisation among asthmatics [27], or no significant effect of asthma on mortality [39]. A systematic review and meta-analysis of 62 studies that included 2 457 205 individuals found a reduced risk of mortality among individuals with asthma [40]. Meta-analysis results were stable and robust, and showed no significant potential publication bias. Another meta-analysis of asthma and COVID-19 outcomes evaluated 51 studies including 379 381 individuals [41]. The risk of COVID-19 infection among asthmatics was reduced compared to individuals without asthma. Differences in hospitalisation, ICU admission and mechanical ventilation between those with and without asthma were not statistically significant. However, this meta-analysis found that Africa and South America were under-represented. We found that asthma lowered the risk of COVID-19 fatality by 1.09-fold in non-indigenous individuals (HR = 0.94; 95% CI = 0.76-0.97), but the HR for asthma in the indigenous group was not significant (HR = 0.90; 95% CI = 0.83-0.97) [40,41]. In the combined analysis, non-indigenous asthmatics had a lowered risk (HR = 0.92) of COVID-19 related fatality, while asthma had no significant effect (*P* = 0.50) among indigenous individuals. We observed that non-indigenous asthmatics individuals were significantly less likely to be hospitalised than indigenous asthmatics. Having asthma did not significantly affect ICU admission or mechanical ventilation. Fatality risk was significantly decreased among non-indigenous asthmatics, but we observed no significant effect of asthma on mortality (**Figure 2**, **Figure 3**, **Figure 4**, **Figure 5**, Tables S1-S4 in the **Online Supplementary Document**).

### Pneumonia

Asymptomatic COVID-19 patients usually becomes better over time without specialised medical care [42]. A meta-analysis of 41 studies comprising 50 155 individuals showed an average of 15.6% (95% CI = 10.1-23.0) of infected patients were asymptomatic [43]. However, symptomatic pneumonia and sudden severe respiratory failure occur among approximately 14% of COVID-19 patients [44], frequently requiring hospitalisation, ICU admission, and mechanical ventilation [45]. In Mexico, COVID-19 patients that developed pneumonia with a history of CKD and were hospitalised had a 30% lower chance of survival after 20 days than those with COVID-19 infection who did not have pneumonia or CKD [46]. COVID-19 patients with pneumonia in our study had significantly higher odds for hospitalisation, admission to ICU, and mechanical ventilation use. COVID-19 fatality HR among indigenous individuals with pneumonia was increased by 1.70-fold. Non-indigenous individuals had an HR of 1.47-fold, with significantly lower pneumonia fatality risk (Table S4 in the **Online Supplementary Document**).

### Hypertension

The prevalence of hypertension among non-indigenous adults in Mexico is 25.5% [47], while 42.7% of the adult Mexican indigenous population had high blood pressure [48]. Among COVID-19 patients, pre-existing hypertension is associated with severe pneumonia, excessive inflammatory reactions, and organ and tissue damage in COVID-19 infection [49]. In Mexico, hypertension significantly increased the HR of COVID-19 fatality 1.24-fold [50]. In the present study, COVID-19 fatality risk for indigenous individuals with hypertension was increased 1.11-fold. Non-indigenous individuals with hypertension had a lower probability of fatality but not significantly so (Table S4 in the **Online Supplementary Document**).

### Cardiovascular

The CVD, including coronary artery disease and heart failure, are the leading causes of death globally [51]. CVD is a major cause of death in Mexico, accounting for 20% of mortality. Viral pneumonia is the main pathology with COVID-19, and this can exacerbate existing cardiovascular disorders (e.g. myocardial injury, arrhythmias, acute cardiac syndrome, thromboembolism) through compromised circulation and oxygenation [52]. COVID-19 patients who present with cardiac symptoms rather than the typical symptoms of fever or cough may be at increased risk of cardiovascular complications [53,54]. Among Mexican COVID-19 cases, patients with CVD and a positive COVID-19 result were 1.15 times more likely to be hospitalised compared to patients who did not have CVD. One study reported no association between CVD diagnosis and COVID-19 fatality [55]. In our study, CVD decreased the risk of COVID-19 fatality for non-indigenous individuals by 7% but had no significant effect on indigenous individuals.

### Smoking

The effect of smoking on COVID-19 outcomes are not consistent across studies. A review of 46 studies indicated the weight of the evidence supported the association of tobacco use (mainly smoking) with more severe immune and inflammatory responses, increased severity of COVID-19 infection [56]. Meta-analysis of the 46 studies of smoking and COVID-19 found that tobacco use had a combined OR of 1.59 (95% CI = 1.39-1.89). Fifteen studies reported an OR of less than 1.0 for smoking and fatality, but none were statistically significant [16,57].

A systematic review and meta-analysis of 109 studies found that smoking was strongly associated with an increased risk of severe or critical (ICU, death) outcomes but mechanical ventilation use was not increased among COVID-19 patients who smoked [58]. In another meta-analysis, current smoking prevalence among those hospitalised for COVID-19 was compared to the smoking prevalence in the general population of China, the USA, and Italy in 720 studies reported significantly lower smoking prevalence among the hospitalised, suggesting a current smoking decreased the likelihood of hospitalisation and mortality [59]. Notably, the study suffers from the ecological inference fallacy because inferences were based upon national population surveys. In our study, smoking significantly decreased the likelihood of COVID-19 fatality by about 1.07-fold (Table S4 in the **Online Supplementary Document**). Notably, other analyses of the Mexico COVID-19 Registry database have published results that smoking reduced fatality risk [60]. The data may contain an unknown that produces the anomalous result with smoking reducing mortality risk in COVID-19 patients.

### Obesity

More than 60% of adults in Mexico are either overweight or obese [61]. Obesity is a major risk factor for adverse outcomes with COVID-19 because of obesity-associated chronic inflammation, reduced immune and blunted treatment responses [62]. We found that obesity significantly increased the risk by 1.21-fold for COVID-19 mortality in indigenous individuals, but less so (1.10-fold) among non-indigenous individuals.

### Immunosuppression (immunocompromised and cancer patients)

Immunosuppression and immunodeficiency are associated with an increased risk of severe COVID-19 disease [63]. Patients with cancer, hematopoietic cell transplant, and those who underwent solid organ transplant are frequently immunocompromised, plus a smaller proportion of heritable immune abnormalities [64]. Higher rates of intensive care and in-hospital COVID-19 mortality were found for immunocompromised patients compared to the general population [65]. In the present study, the association of fatality risk with being immunocompromised was not different between non-indigenous (1.11-fold) and indigenous individuals (1.07 fold).

### CKD

Mexico has no national renal registry, limiting the epidemiology of CKD and ESRD. CKD is associated with poorer outcomes in patients with COVID-19 compared to those without CKD [14,66]. The prevalence of CKD in Mexico is 20%-33% of the population, and somewhat higher among indigenous people [67-69]. CKD was an independent risk factor for more severe COVID-19 disease presentation and mortality after adjusting for other comorbidities [70]. We found that CKD significantly increased the risk of COVID-19 mortality, but non-indigenous and indigenous individuals did not significantly differ significantly (1.4- and 1.3-fold, respectively).

Our study adds to the body of evidence of the disease burden faced by marginalised indigenous communities in Mexico during the COVID-19 pandemic, indicating that indigenous and marginalised individuals were at a higher risk of hospitalisation. While asthma was protective against hospitalisation in non-indigenous people, it increased the risk of hospitalisation in indigenous people.

### Summary

Indigenous marginalised individuals had significantly lower probability of ICU admission compared to non-indigenous marginalised individuals, who were more likely to be admitted to an ICU unit. Among the indigenous marginalised individuals, the probability of mechanical ventilation was significantly reduced compared to non-indigenous marginalised individuals, indigenous individuals with diabetes, pneumonia, hypertension, and obesity were at a higher risk of COVID-19 related mortality compared to non-indigenous individuals. CVD, ICU admission, days to hospitalisation, smoking, and asthma decreased the likelihood of COVID-19 fatality (Table S5 in the **Online Supplementary Document**).

### Limitations

#### Sample bias

Although the sample size is large, the data set does not provide information on disease outcomes/specification and socioeconomic status, i.e. occupation and income. As this is an observational study, we could not determine causality [71]. However, the greatest limitation of our study is the under sampling of indigenous individuals. Using the 2020 census, the sample should have contained 15 times more indigenous individuals than it does. In 2020, there were 112 236 538 individuals in the Mexico Census, and 16 933 285 indigenous individuals (15.3%). Our sample included 32 211 indigenous individuals, comprising 0.98% of the total sample. We do not have an explanation for why the under sampling occurred, and we do not know what type of bias this introduces.

#### Results cannot be extrapolated

These COVID-19 registry data are specific to Mexico and may not be extrapolated to other nations because of the uniqueness of the indigenous populations. Other Latin American countries are different from Mexico in culture, economics, and indigenous cultures. Importantly, proximity to the USA also precludes extrapolation to other countries, as Mexico is influenced more by the USA than any other Latin American country. African race outcomes are not in the database, and the smoking analysis yielded suspect results that seem biologically implausible.

#### Marginalisation

The marginalisation index indirectly captures the socioeconomic status of the study participants, but integrates isolation by distance into the index, making it impossible to point to ascribed specific distance effects. The use of the index is another limitation because we had only *municipio* level index values, leading to ecological inference fallacy. In summary, the major study limitations include the observational nature of the study in which secondary data from the Mexico COVID-19 Registry was analysed, which prevents conclusions regarding causality, the absence of socioeconomic status, occupation, and distance from residence to hospital from the data (with the latter being a known social determinant of health that strongly affects clinical outcomes), and the limited clinical relevance of our findings.

## CONCLUSIONS

We found that COVID-19 positive marginalised indigenous populations are at higher risk of hospitalisation and admission to ICU units compared to non-indigenous and non-marginalised populations in Mexico. Despite the higher risk, marginalised indigenous populations are less likely to be placed on mechanical ventilation. In the interaction model, marginalised indigenous individuals were less likely to be admitted to the ICU, and it was less likely that they would be placed in ventilation. However, marginalised indigenous individuals were about as likely to die as the non-indigenous patients were. Additional evidence indicates that asthma, CVD, smoking, ICU admission, and increase in days to hospitalisation are increased the probability COVID-19 related fatality would not occur. Risk factors such as diabetes, pneumonia, hypertension, and obesity increased the risk of COVID-19 related fatality among indigenous individuals compared to non-indigenous individuals. A possible mitigation for future pandemics may be to provide increased access to ICUs and mechanical ventilation in rural hospitals, where a large proportion of indigenous people are hospitalised.

## Additional material


Online Supplementary Document
