# Supplementary material for: COVID-19 infection and mortality among non-pregnant indigenous adults in Mexico 2020-2022: Impact of marginalisation
Source: J Glob Health. 2023 Jul 28;13:06030. doi: 10.7189/jogh.13.06030 (PMC10386760; doi:10.7189/jogh.13.06030)
Supplement: Online Supplementary Document [file jogh-13-06030-s001.pdf]

Supplemental Materials

Table S1. Supplemental Logistic Regression Analysis of Hospitalization
Table S2. Supplemental Logistic Regression Analysis of Intensive Care Unit (ICU) Admission
Table S3. Supplemental Logistic Regression Analysis of Mechanical Ventilation
Table S4. Cox Regression Analysis of Mortality
Table S5. Indigenous Status, Marginalized + Ind × Marg Interaction: Combined Sample
Table S6. Distribution of Missing Values in the Mexico COVID-19 Registry Variables used in the Present Study
Table S7. Unadjusted ORs and HRs Predicting Hospitalization, ICU Admission, Ventilator Use, and Fatality by Indigenous Status

Table S1. Supplemental Logistic Regression Analysis of Hospitalization

Variable	Combined (N = 3283022) OR	Lower 95% CI	Upper 95% CI	P	Non-Indigenous (N = 3250946) OR	Lower 95% CI	Upper 95% CI	P	Indigenous (N = 32076) OR	Lower 95% CI	Upper 95% CI	P
Age	2.36	2.28	2.44	0.00	2.36	2.28	2.44	0.00	2.43	1.83	3.24	0.00
Sex (ref: female)	1.53	1.51	1.55	0.00	1.54	1.52	1.55	0.00	1.34	1.21	1.49	0.00
Diabetes	2.94	2.89	2.99	0.00	2.94	2.89	2.99	0.00	2.92	2.55	3.35	0.00
COPD	1.90	1.78	2.04	0.00	1.90	1.77	2.03	0.00	2.39	1.52	3.79	0.00
Asthma	0.94	0.90	0.98	0.00	0.94	0.90	0.98	0.00	1.05	0.74	1.47	0.80
Pneumonia	99.23	97.88	100.58	0.00	99.44	98.08	100.81	0.00	84.67	75.32	95.17	0.00
Hypertension	1.89	1.86	1.93	0.00	1.90	1.87	1.93	0.00	1.45	1.25	1.67	0.00
Cardiovascular	1.60	1.52	1.68	0.00	1.60	1.52	1.69	0.00	1.25	0.79	1.98	0.35
Obesity	1.57	1.55	1.59	0.00	1.57	1.54	1.59	0.00	1.64	1.44	1.86	0.00
CKD	5.85	5.63	6.08	0.00	5.89	5.66	6.12	0.00	3.25	2.21	4.77	0.00
Immunosuppressed	2.33	2.20	2.47	0.00	2.34	2.21	2.48	0.00	1.56	0.91	2.64	0.10
Smoking	0.81	0.79	0.83	0.00	0.81	0.79	0.83	0.00	0.76	0.60	0.97	0.03
Marginalization	1.16	1.14	1.19	0.00	1.17	1.14	1.20	0.00	1.13	1.02	1.25	0.02
Indigenous	1.24	1.18	1.31	0.00								

Combined: Sensitivity = 96.42%, Specificity = 80.83%
[1]Hosmer- Lemeshow χ^2 = 14.2 , p = .014

Non-Indigenous: Sensitivity = 96.43%, Specificity = 80.78%.
Hosmer- Lemeshow χ^2 = 9.5, p = 0.091

Indigenous: Sensitivity = 94.64%, Specificity = 84.37%
Hosmer- Lemeshow χ^2 = 10.4, p = .063

[1]All Hosmer-Lemeshow tests are conducted on random samples of ~25,000 individuals

Table S2. Supplemental Logistic Regression Analysis of Intensive Care Unit (ICU) Admission

	Combined (N = 3283022)				Non-Indigenous (N = 3250946)				Indigenous (N = 32076)			
Variable	OR	Lower 95% CI	Upper 95% CI	P	OR	Lower 95% CI	Upper 95% CI	P	OR	Lower 95% CI	Upper 95% CI	P
Age	1.25	1.17	1.34	0.00	1.25	1.16	1.34	0.00	1.34	0.85	2.11	0.20
Sex (ref: female)	1.40	1.36	1.45	0.00	1.40	1.36	1.45	0.00	1.50	1.20	1.88	0.00
Diabetes	1.37	1.32	1.42	0.00	1.37	1.32	1.42	0.00	1.27	0.99	1.63	0.06
COPD	1.08	0.95	1.23	0.23	1.07	0.94	1.22	0.32	1.57	0.83	2.96	0.17
Asthma	1.03	0.93	1.13	0.60	1.02	0.93	1.13	0.67	1.22	0.62	2.40	0.56
Pneumonia	75.64	72.52	78.90	0.00	76.01	72.85	79.31	0.00	51.73	37.44	71.48	0.00
Hypertension	1.06	1.02	1.10	0.00	1.06	1.02	1.10	0.00	0.91	0.69	1.19	0.48
Cardiovascular	1.23	1.12	1.36	0.00	1.24	1.12	1.37	0.00	1.02	0.47	2.21	0.95
Obesity	1.56	1.51	1.61	0.00	1.56	1.51	1.61	0.00	1.57	1.25	1.99	0.00
CKD	0.83	0.76	0.90	0.00	0.83	0.76	0.90	0.00	0.81	0.44	1.51	0.51
Immunosuppressed	1.36	1.22	1.51	0.00	1.35	1.22	1.51	0.00	1.50	0.69	3.25	0.31
Smoking	0.87	0.82	0.92	0.00	0.87	0.82	0.92	0.00	0.74	0.46	1.20	0.22
Marginalization	1.20	1.14	1.27	0.00	1.24	1.17	1.31	0.00	0.79	0.64	0.98	0.03
Indigenous	1.22	1.09	1.36	0.00								

Combined: Sensitivity = 99.31%, Specificity = Null; [1]Hosmer- Lemeshow χ^2 = 3.0, p = .556

Non-Indigenous: Sensitivity = 99.32%, Specificity = Null; Hosmer- Lemeshow χ^2 = 1.7, p = .785

Indigenous: Sensitivity = 98.44%, Specificity = Null; Hosmer- Lemeshow χ^2 = 2.4, p = .792

[1]All Hosmer-Lemeshow tests are conducted on random samples of ~25,000 individuals

Table S3. Supplemental Logistic Regression Analysis of Mechanical Ventilation

	Combined (N = 465509)				Non-Indigenous (N = 457795)				Indigenous (N = 7714)			
**Variable**	**OR**	**Lower**	**Upper**	**P**	**OR**	**Lower**	**Upper**	**P**	**OR**	**Lower**	**Upper**	**P**
		**95% CI**				**95% CI**				**95% CI**		
Age	1.19	1.13	1.26	0.00	1.01	1.01	1.01	0.00	1.49	1.00	2.22	0.05
Sex (ref: female)	1.32	1.29	1.36	0.00	1.26	1.23	1.28	0.00	1.26	1.02	1.56	0.03
Diabetes	1.11	1.08	1.15	0.00	1.04	1.02	1.06	0.00	1.02	0.81	1.29	0.86
COPD	1.05	0.95	1.17	0.31	0.93	0.88	0.98	0.00	1.02	0.53	1.96	0.95
Asthma	1.07	0.99	1.17	0.10	1.09	1.02	1.16	0.01	1.14	0.59	2.19	0.71
Pneumonia	3.73	3.61	3.86	0.00	3.53	3.44	3.61	0.00	3.13	2.34	4.18	0.00
Hypertension	1.15	1.12	1.19	0.00	1.09	1.07	1.11	0.00	0.94	0.73	1.22	0.65
Cardiovascular	1.16	1.07	1.26	0.00	1.03	0.98	1.07	0.25	0.54	0.23	1.29	0.17
Obesity	1.42	1.38	1.46	0.00	1.39	1.36	1.41	0.00	1.58	1.27	1.97	0.00
CKD	0.98	0.93	1.04	0.55	0.95	0.91	0.98	0.01	1.15	0.70	1.88	0.59
Immunosuppressed	1.27	1.16	1.38	0.00	1.24	1.16	1.31	0.00	1.80	0.91	3.57	0.09
Smoking	0.99	0.94	1.04	0.68	1.00	0.97	1.04	0.95	1.02	0.67	1.56	0.92
Marginalization	0.92	0.87	0.97	0.00	0.92	0.89	0.95	0.00	0.58	0.48	0.72	0.00
Indigenous	0.97	0.88	1.08	0.59								

Combined: Sensitivity = 88.53%, Specificity = Null
[1]Hosmer- Lemeshow χ^2 = 1.13, p = .992

Non-Indigenous: Sensitivity = 88.53%, Specificity = Null
Hosmer- Lemeshow χ^2 = 8.79, p = .360

Indigenous: Sensitivity = 88.33%, Specificity = Null
Hosmer- Lemeshow χ^2 = 6.39, p = .604

[1]All Hosmer-Lemeshow tests are conducted on random samples of ~25,000 individuals

Table S4. Cox Regression analysis of mortality

	Combined (N = 465509)				Non-Indigenous (N = 457795)				Indigenous (N = 7714)			
**Variable**	**HR**	**95% CI**		**P**	**HR**	**95% CI**		**P**	**HR**	**95% CI**		**P**
		**Lower**	**Upper**			**Lower**	**Upper**			**Lower**	**Upper**	
Age	1.03	1.03	1.03	0.00	1.03	1.03	1.03	0.00	1.02	1.02	1.03	0.00
Sex (ref: female)	1.16	1.15	1.17	0.00	1.16	1.15	1.17	0.00	1.14	1.06	1.22	0.00
Diabetes	1.12	1.11	1.13	0.00	1.12	1.11	1.13	0.00	1.18	1.09	1.26	0.00
COPD	1.00	0.98	1.02	0.83	1.01	0.98	1.03	0.66	0.92	0.80	1.05	0.22
Asthma	0.92	0.89	0.95	0.00	0.92	0.89	0.95	0.00	1.08	0.87	1.33	0.50
Pneumonia	1.47	1.46	1.49	0.00	1.47	1.45	1.48	0.00	1.70	1.56	1.84	0.00
Hypertension	1.09	1.08	1.10	0.00	1.09	1.08	1.10	0.00	1.11	1.03	1.19	0.01
Cardiovascular	0.93	0.91	0.95	0.00	0.93	0.91	0.95	0.00	1.01	0.86	1.19	0.92
Obesity	1.11	1.09	1.12	0.00	1.10	1.09	1.12	0.00	1.21	1.11	1.30	0.00
CKD	1.40	1.38	1.43	0.00	1.40	1.38	1.43	0.00	1.30	1.11	1.51	0.00
Immunosuppressed	1.11	1.07	1.14	0.00	1.11	1.07	1.14	0.00	1.13	0.88	1.44	0.34
Smoking	0.93	0.91	0.94	0.00	0.93	0.91	0.94	0.00	0.84	0.73	0.97	0.02
Mechanical Ventilation	2.52	2.49	2.55	0.00	2.52	2.49	2.55	0.00	2.36	2.16	2.58	0.00
ICU Admission	0.84	0.82	0.85	0.00	0.84	0.82	0.85	0.00	0.90	0.81	1.00	0.04
Days to Hospitalization	0.97	0.97	0.98	0.00	0.97	0.97	0.98	0.00	0.98	0.97	0.99	0.00
Marginalization	1.00	0.98	1.02	0.89	1.00	0.98	1.01	0.57	1.07	1.00	1.14	0.05
Indigenous	1.04	1.00	1.07	0.05								

Table S5. Indigenous Status, Marginalized + Ind × Marg Interaction: Combined Sample

	Indigenous				Marginalized				Indigenous × Marginalized			
	OR	95% CI				95% CI				95% CI		
		Lower	Upper	P-Value	OR	Lower	Upper	P-Value	OR	Lower	Upper	P-Value
**Hospitalization:**												
**Linear**	1.15	1.11	1.2	0.00	1.05	1.03	1.07	0.00				
**Interaction**	1.16	1.09	1.24	0.00	1.05	1.03	1.07	0.00	0.98	0.90	1.07	0.69
**ICU:**												
**Linear**	1.17	1.09	1.27	0.00	1.23	1.19	1.28	0.00				
**Interaction**	1.50	1.36	1.67	0.00	1.28	1.26	1.33	0.00	0.60	0.52	0.71	0.00
**Mechanical Ventilation:**												
**Linear**	0.94	0.88	1.01	0.09	0.89	0.86	0.92	0.00				
**Interaction**	1.15	1.05	1.26	0.00	0.92	0.89	0.95	0.00	0.64	0.55	0.74	0.00
**Death:**												
**Linear**	1.04	1.00	1.07	0.05	1.00	0.98	1.02	0.89				
**Interaction**	1.01	0.96	1.05	0.85	1.00	0.99	1.01	0.56	1.06	1.00	1.14	0.09

1. **For Death, OR is expressing hazard ratio in the table.**
2. **The odds/hazard ratios are modelled controlling for same covariates in the linear models.**

Table S6. Distribution of Missing Values in the Mexico COVID-19 Registry Variables used in the Present Study

Variable	Total (N = 3,424,690)			Indigenous (N = 32,211)			Non-Indigenous (N = 3,261,685)		
	N	N complete data (%)	N missing (%)	N	Complete data (%)	N missing (%)	N	Complete data (%)	N missing (%)
Age	3,424,690	100	0	32,211	100	0	3,261,685	100	0
Sex	3,424,690	100	0	32,211	100	0	3,261,685	100	0
Diabetes	3,416,515	99.8	0.2	32,152	99.8	0.2	3,254,350	99.8	0.2
COPD	3,417,156	99.8	0.2	32,162	99.8	0.2	3,254,955	99.8	0.2
Asthma	3,417,272	99.8	0.2	32,164	99.9	0.1	3,255,049	99.8	0.2
Pneumonia	3,419,997	99.9	0.1	32,210	100	0	3,261,659	100	0
Hypertension	3,417,021	99.8	0.2	32,166	99.9	0.1	3,254,796	99.8	0.2
Cardiovascular	3,417,141	99.8	0.2	32,161	99.8	0.2	3,254,915	99.8	0.2
Obesity	3,417,396	99.8	0.2	32,168	99.9	0.1	3,255,157	99.8	0.2
CKD	3,417,209	99.8	0.2	32,156	99.8	0.2	3,254,992	99.8	0.2
Immunosuppressed	3,417,112	99.8	0.2	32,163	99.9	0.1	3,254,879	99.8	0.2
Smoking	3,416,878	99.8	0.2	32,156	99.8	0.2	3,254,697	99.8	0.2
Marginalization	3,424,651	100	0	32,197	100	0	3,261,661	100	0

Little's statistic for MCR = xx.xx, p < 0.xxx

Table S7. Unadjusted ORs and HRs predicting hospitalization, ICU admission, ventilator use, and fatality by indigenous status

	Hospitalization			ICU admission			Mechanical ventilation			Fatality		
	uOR	95% CI		uOR	95% CI		uOR	95% CI		uHR	95% CI	
		Lower	Upper		Lower	Upper		Lower	Upper		Lower	Upper
Age	1.08	1.08	1.08	1.06	1.06	1.06	1.01	1.01	1.01	1.08	1.07	1.08
Sex (ref: female)	1.56	1.55	1.57	1.74	1.71	1.78	1.23	1.21	1.26	1.66	1.65	1.67
Diabetes	5.75	5.71	5.79	4.18	4.09	4.27	1.14	1.12	1.17	5.19	5.15	5.24
COPD	8.13	7.95	8.32	3.94	3.72	4.17	1.05	1.00	1.10	6.05	5.93	6.17
Asthma	1.03	1.01	1.06	1.03	0.96	1.11	1.07	1.01	1.13	0.92	0.89	0.95
Pneumonia	108.35	107.19	109.51	62.73	60.86	64.65	3.67	3.59	3.76	28.67	28.41	28.93
Hypertension	5.00	4.97	5.04	3.59	3.52	3.67	1.20	1.18	1.22	5.13	5.08	5.17
Cardiovascular	5.97	5.85	6.08	4.12	3.93	4.33	1.17	1.13	1.22	4.84	4.75	4.93
Obesity	2.15	2.13	2.17	2.60	2.54	2.66	1.43	1.40	1.45	2.06	2.04	2.08
CKD	11.26	11.04	11.50	3.68	3.49	3.87	0.99	0.95	1.03	7.58	7.46	7.71
Immunosuppressed	4.61	4.49	4.74	3.54	3.29	3.80	1.25	1.18	1.32	3.80	3.70	3.91
Smoking	1.16	1.14	1.17	1.12	1.08	1.17	1.12	1.09	1.16	1.15	1.14	1.17
Marginalization	1.54	1.52	1.56	1.81	1.75	1.88	0.94	0.91	0.97	1.49	1.47	1.52
Indigenous	1.94	1.89	1.99	2.28	2.12	2.45	0.97	0.90	1.03	1.87	1.81	1.93
Mechanical Ventilation										2.73	2.70	2.76
ICU Admission										12.14	11.97	12.31
Days to Hospitalization										1.02	1.02	1.02

Supplemental Figures

Figure S1. Non-indgenous and Indigenous by Sex Survival Graphs
Figure S2. Non-indgenous and Indigenous by Diabetes Survival Graphs
Figure S3. Non-indgenous and Indigenous by COPD Survival Graphs
Figure S4. Non-indgenous and Indigenous by Asthma Survival Graphs
Figure S5. Non-indgenous and Indigenous by Penumonia Survival Graphs
Figure S6. Non-indgenous and Indigenous by Hypertension Survival Graphs
Figure S7. Non-indgenous and Indigenous by Cardiovascular Disease Survival Graphs
Figure S8. Non-indgenous and Indigenous by Obesity Survival Graphs
Figure S9. Non-indgenous and Indigenous by Chronic Kidney Disease Survival Graphs
Figure S10. Non-indgenous and Indigenous by Immunusupression Survival Graphs
Figure S11. Non-indgenous and Indigenous by Smoking Survival Graphs
Figure S12. Non-indgenous and Indigenous by Marginalisation (top 75%) Survival Graphs
Figure S13. Non-indgenous and Indigenous by ICU Admission Survival Graphs
Figure S14. Non-indgenous and Indigenous Mechanical Ventilation Survival Graphs

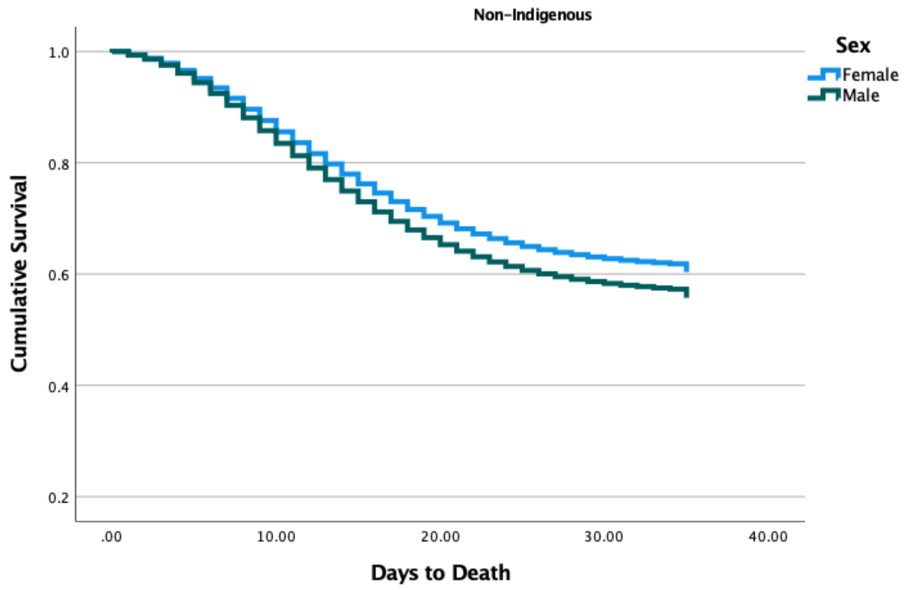
Non-Indigenous
Sex
Female
Male
Cumulative Survival
1.0
0.8
0.6
0.4
0.2
.00
10.00
20.00
30.00
40.00
Days to Death

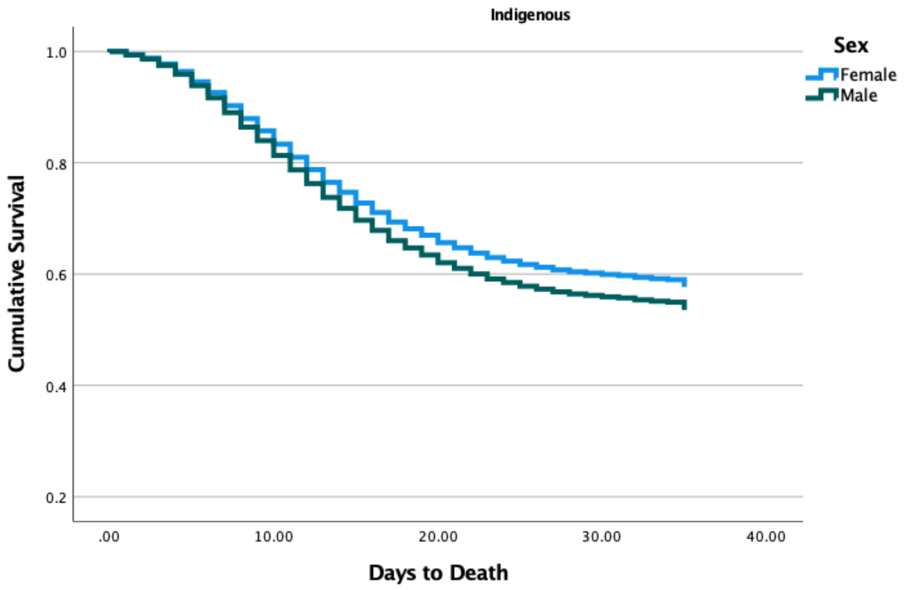
Indigenous
Sex
Female
Male
Cumulative Survival
1.0
0.8
0.6
0.4
0.2
.00
10.00
20.00
30.00
40.00
Days to Death

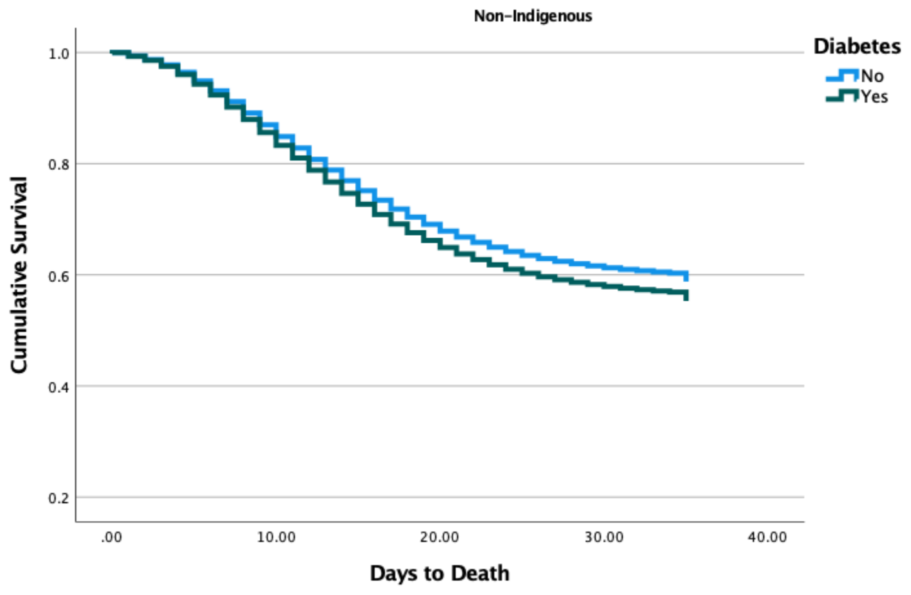
Non-Indigenous
Diabetes
No
Yes
Cumulative Survival
1.0
0.8
0.6
0.4
0.2
.00
10.00
20.00
30.00
40.00
Days to Death

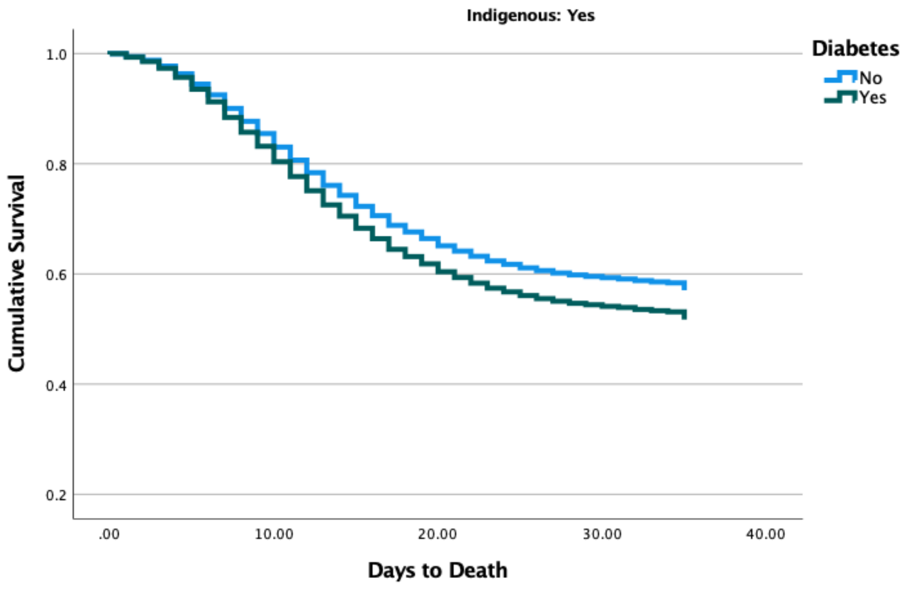
Indigenous: Yes
Diabetes
No
Yes
Cumulative Survival
1.0
0.8
0.6
0.4
0.2
.00
10.00
20.00
30.00
40.00
Days to Death

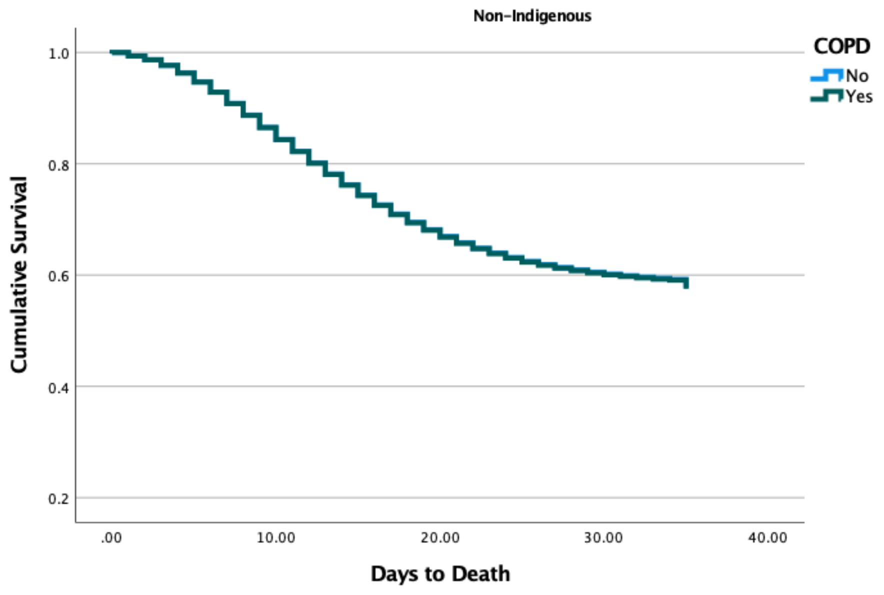
Non-Indigenous
Cumulative Survival
1.0
0.8
0.6
0.4
0.2
.00
10.00
20.00
30.00
40.00
Days to Death
COPD
No
Yes

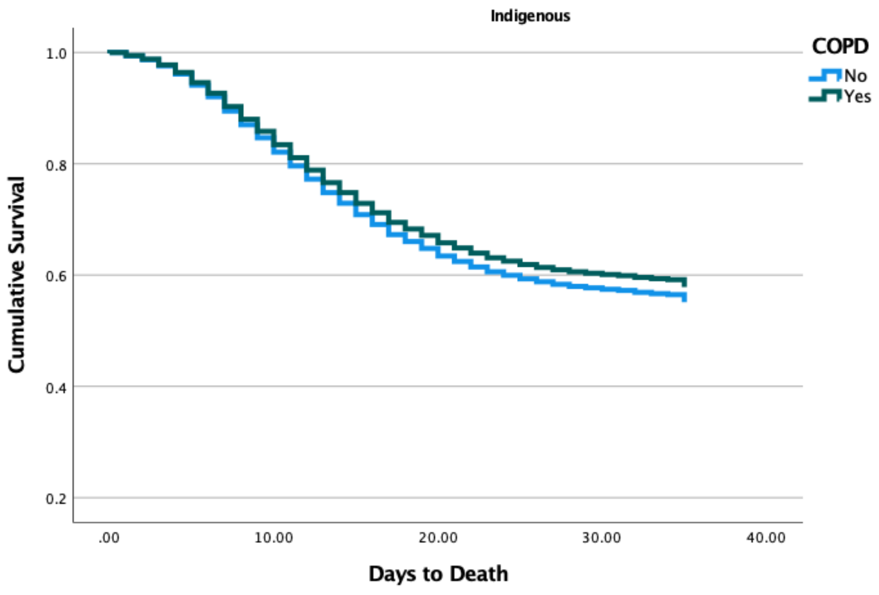
Indigenous
Cumulative Survival
1.0
0.8
0.6
0.4
0.2
.00
10.00
20.00
30.00
40.00
Days to Death
COPD
No
Yes

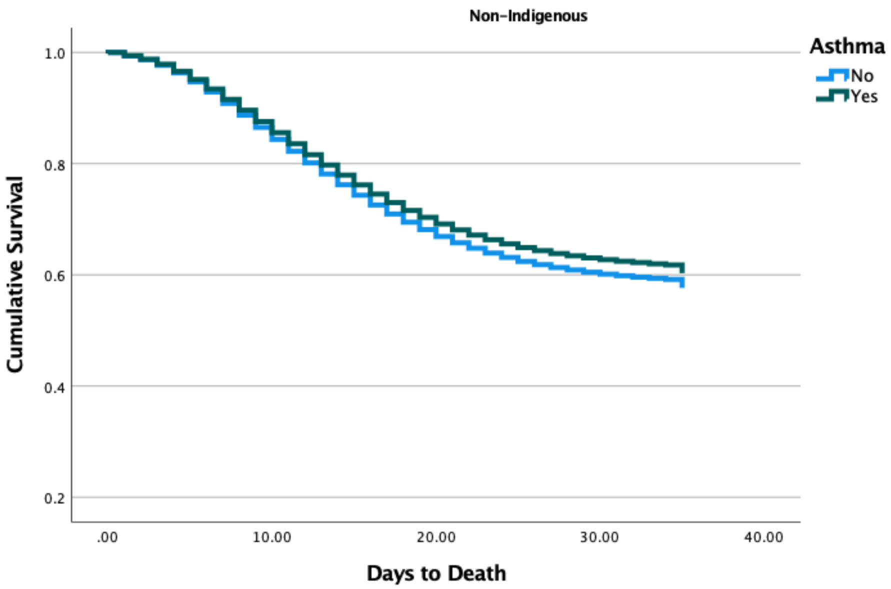
Non-Indigenous
Asthma
No
Yes
Cumulative Survival
1.0
0.8
0.6
0.4
0.2
.00
10.00
20.00
30.00
40.00
Days to Death

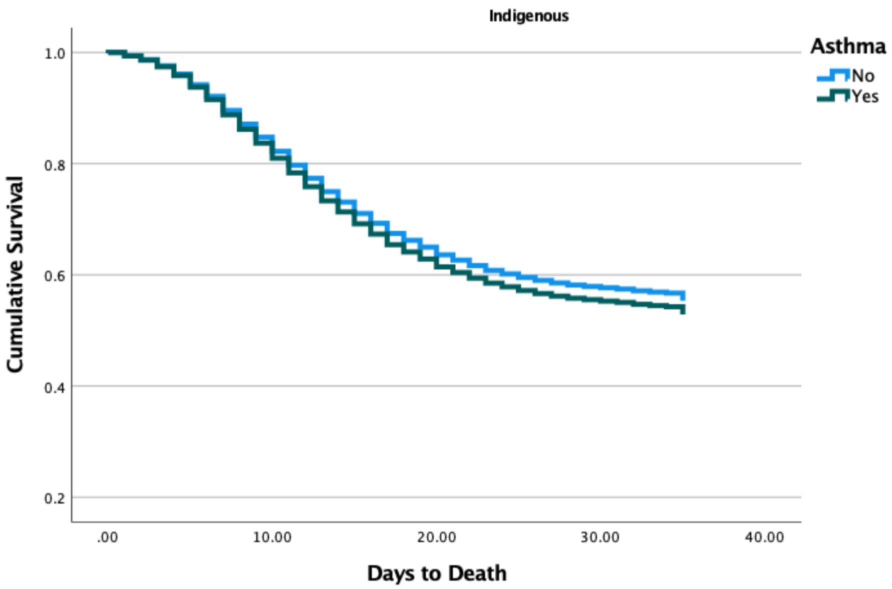
Indigenous
Asthma
No
Yes
Cumulative Survival
1.0
0.8
0.6
0.4
0.2
.00
10.00
20.00
30.00
40.00
Days to Death

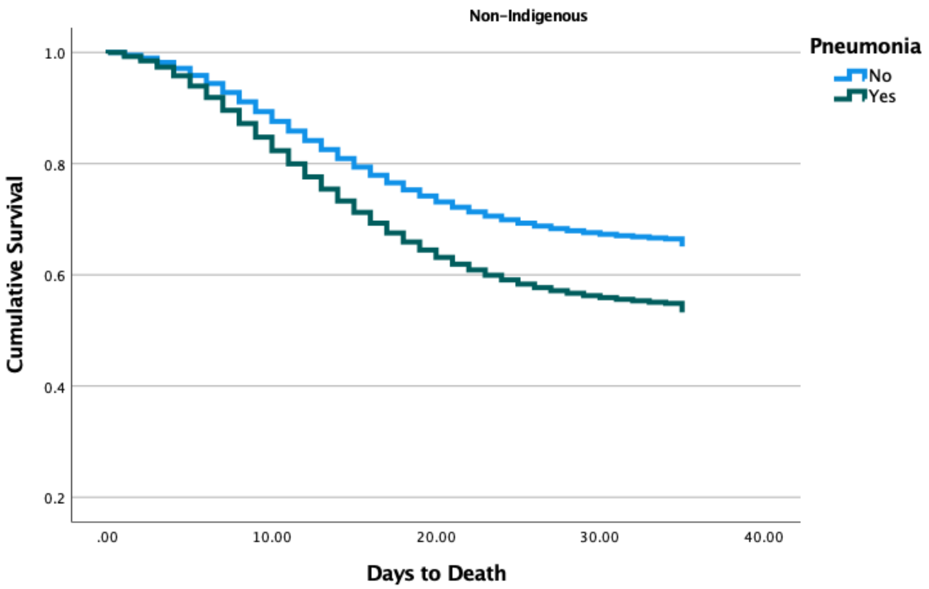
Non-Indigenous
Pneumonia
No
Yes
Cumulative Survival
1.0
0.8
0.6
0.4
0.2
.00
10.00
20.00
30.00
40.00
Days to Death

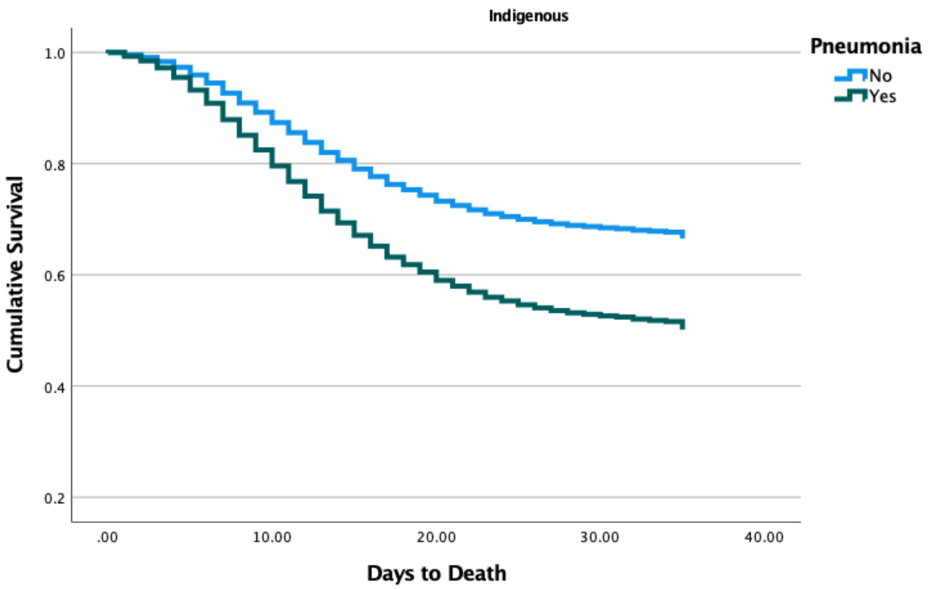
Indigenous
Pneumonia
No
Yes
Cumulative Survival
1.0
0.8
0.6
0.4
0.2
.00
10.00
20.00
30.00
40.00
Days to Death

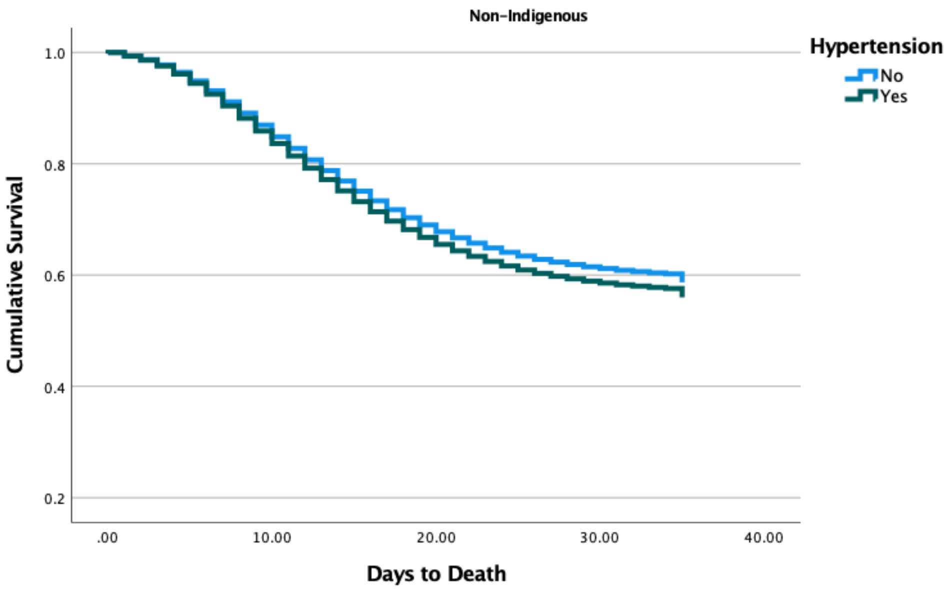
Non-Indigenous
Hypertension
No
Yes
Cumulative Survival
1.0
0.8
0.6
0.4
0.2
.00
10.00
20.00
30.00
40.00
Days to Death

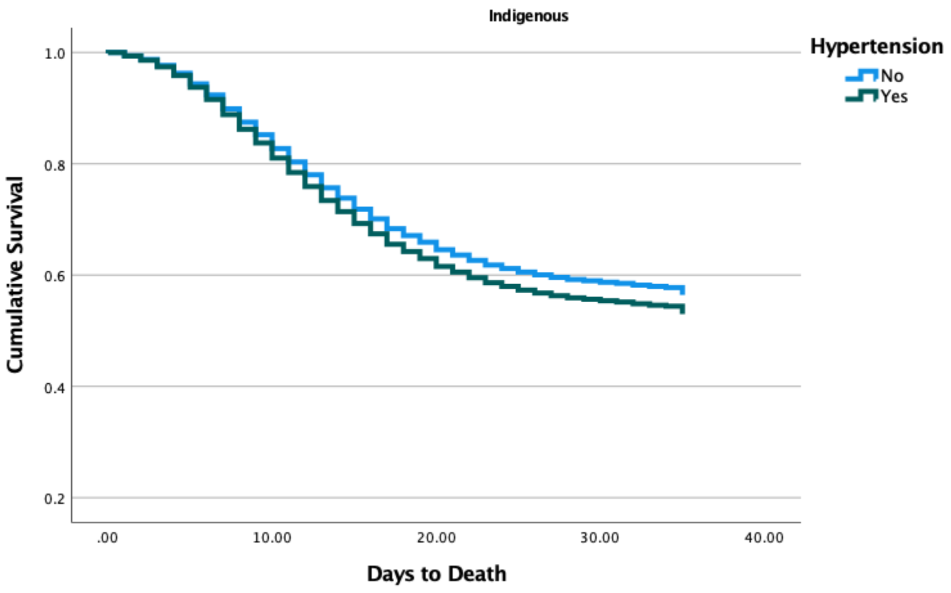
Indigenous
Hypertension
No
Yes
Cumulative Survival
1.0
0.8
0.6
0.4
0.2
.00
10.00
20.00
30.00
40.00
Days to Death

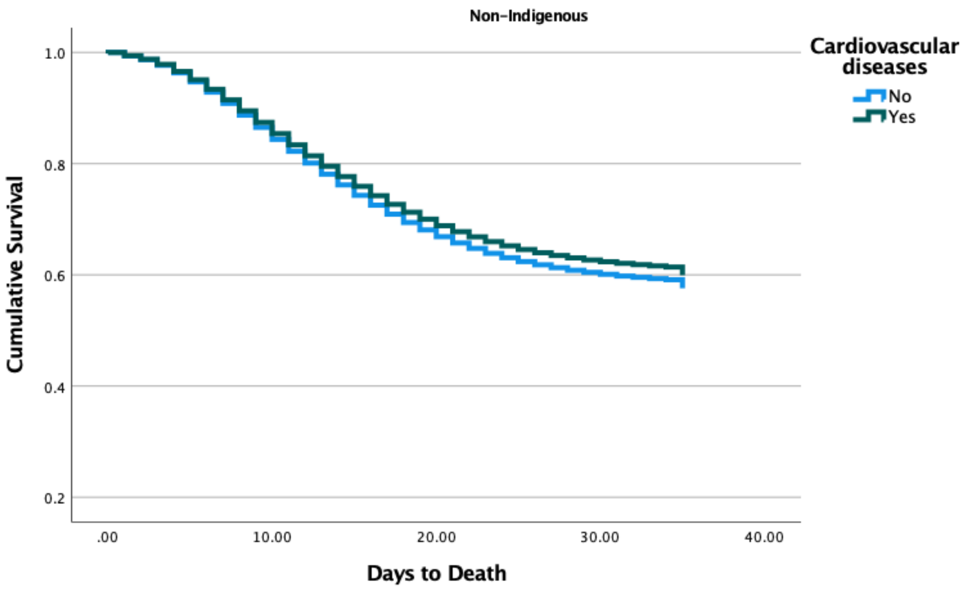
Non-Indigenous
Cumulative Survival
1.0
0.8
0.6
0.4
0.2
.00
10.00
20.00
30.00
40.00
Days to Death
Cardiovascular diseases
No
Yes

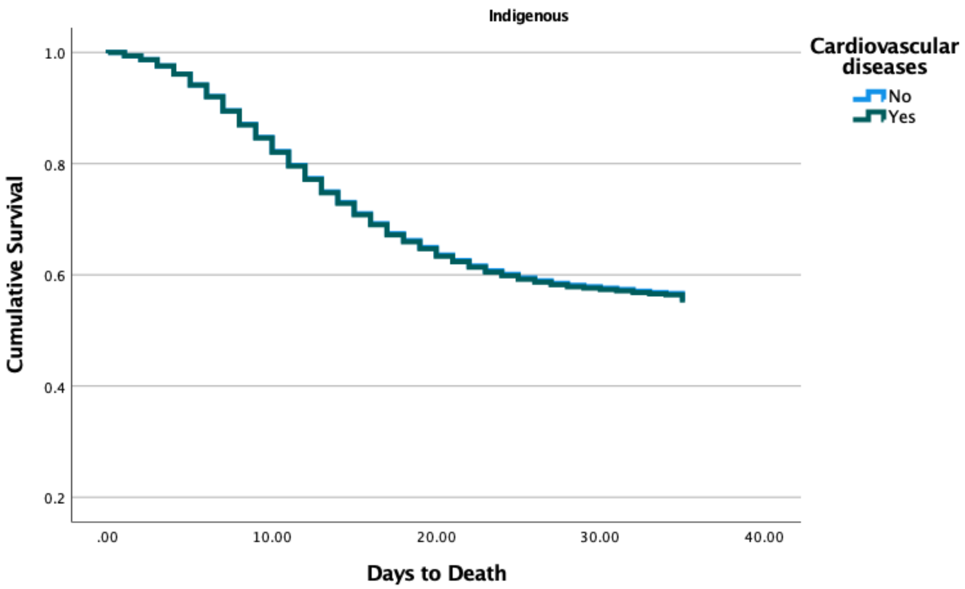
Indigenous
Cumulative Survival
1.0
0.8
0.6
0.4
0.2
.00
10.00
20.00
30.00
40.00
Days to Death
Cardiovascular diseases
No
Yes

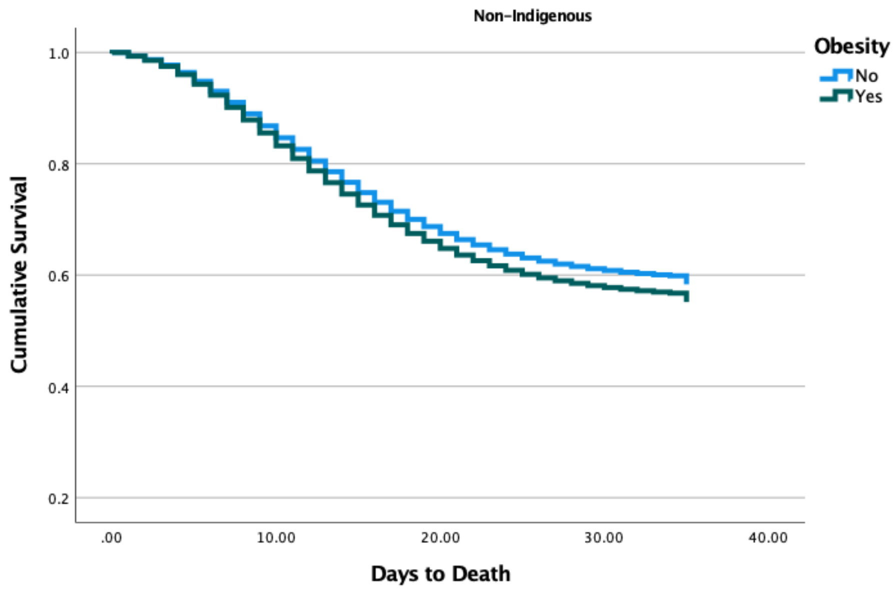
Non-Indigenous
Obesity
No
Yes
Cumulative Survival
1.0
0.8
0.6
0.4
0.2
.00
10.00
20.00
30.00
40.00
Days to Death

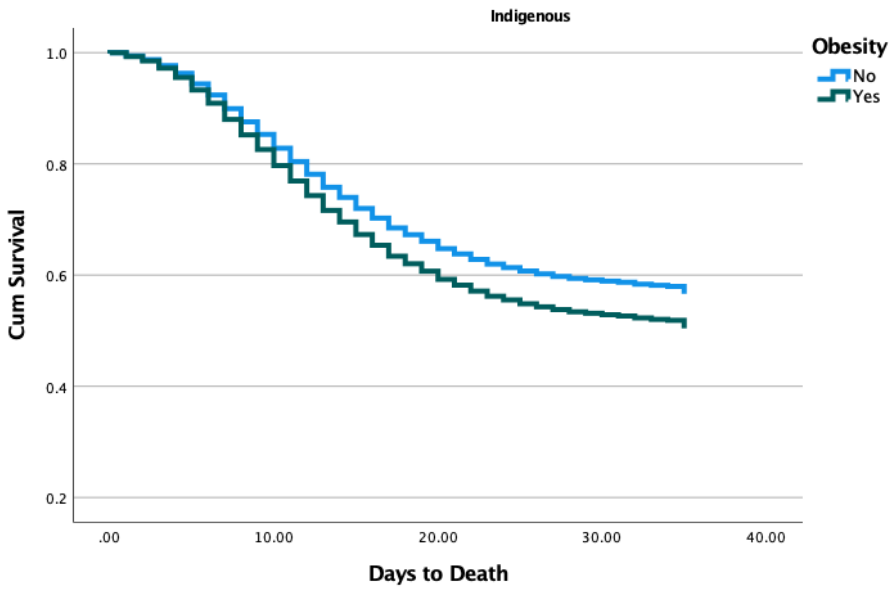
Indigenous
Obesity
No
Yes
Cum Survival
1.0
0.8
0.6
0.4
0.2
.00
10.00
20.00
30.00
40.00
Days to Death

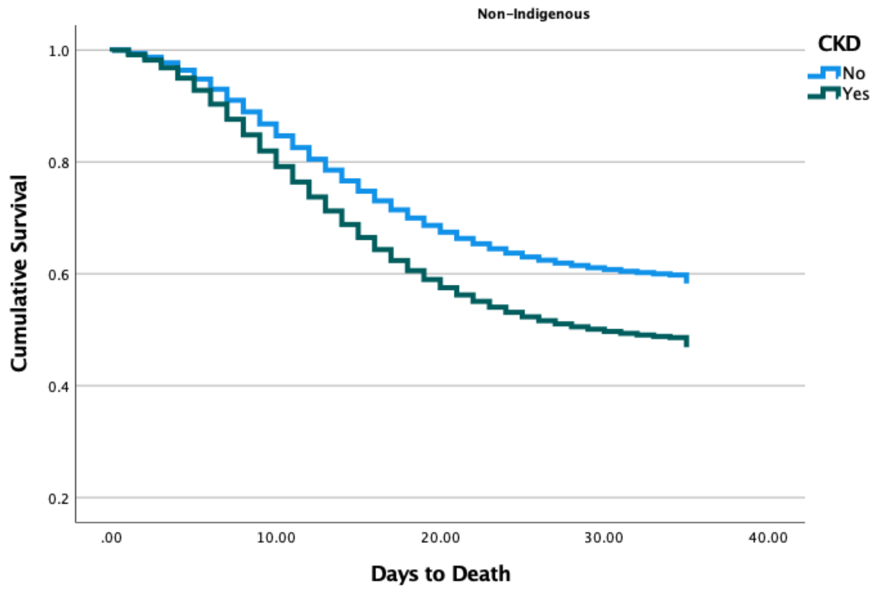
Non-Indigenous
CKD
No
Yes
Cumulative Survival
1.0
0.8
0.6
0.4
0.2
.00
10.00
20.00
30.00
40.00
Days to Death

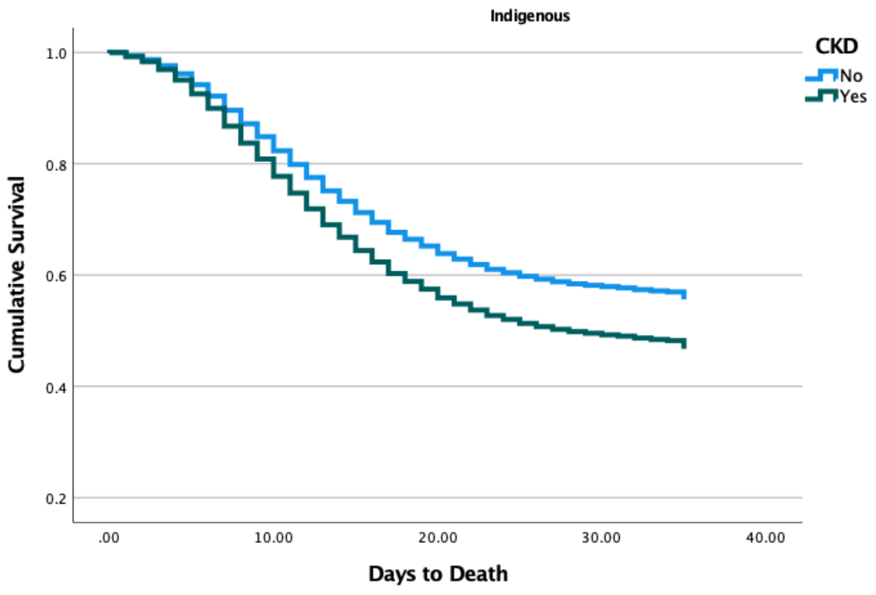
Indigenous
CKD
No
Yes
Cumulative Survival
1.0
0.8
0.6
0.4
0.2
.00
10.00
20.00
30.00
40.00
Days to Death

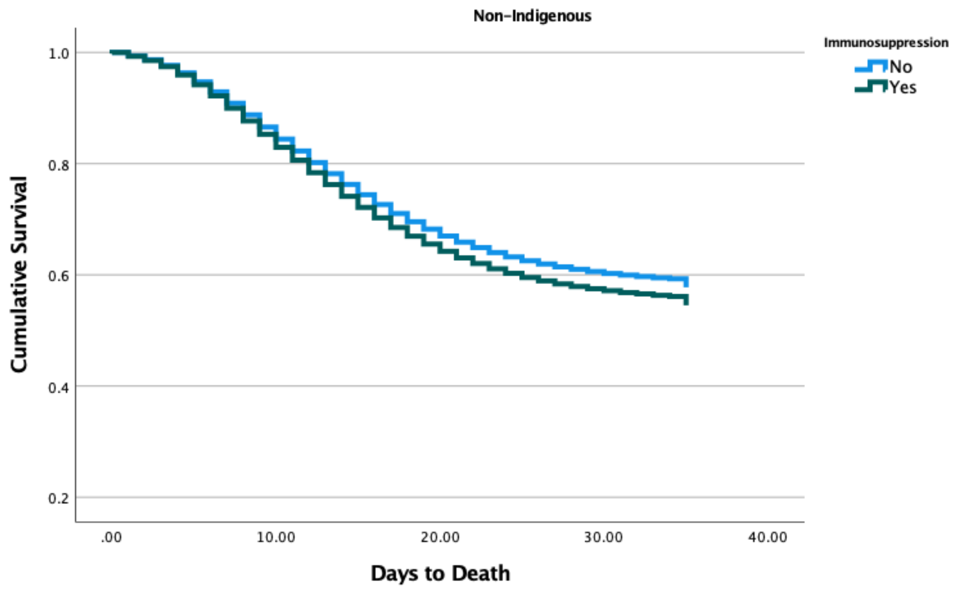
Non-Indigenous
Immunosuppression
No
Yes
Cumulative Survival
1.0
0.8
0.6
0.4
0.2
.00
10.00
20.00
30.00
40.00
Days to Death

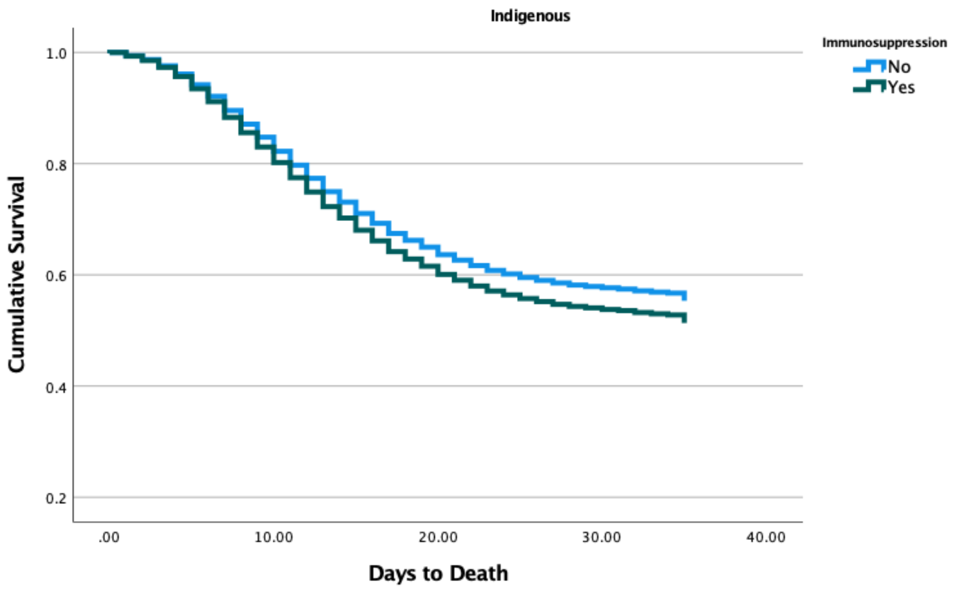
Indigenous
Immunosuppression
No
Yes
Cumulative Survival
1.0
0.8
0.6
0.4
0.2
.00
10.00
20.00
30.00
40.00
Days to Death

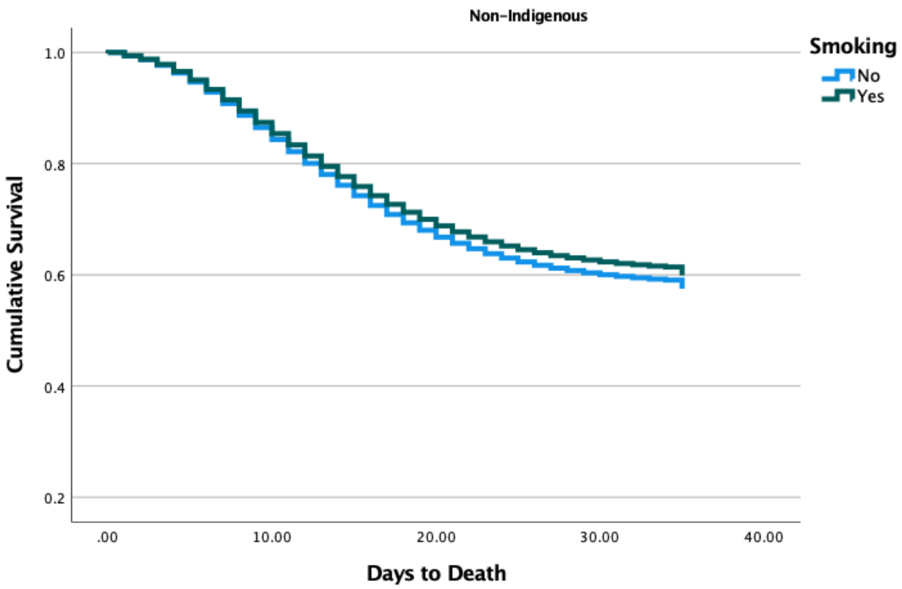
Non-Indigenous
Smoking
No
Yes
Cumulative Survival
1.0
0.8
0.6
0.4
0.2
.00
10.00
20.00
30.00
40.00
Days to Death

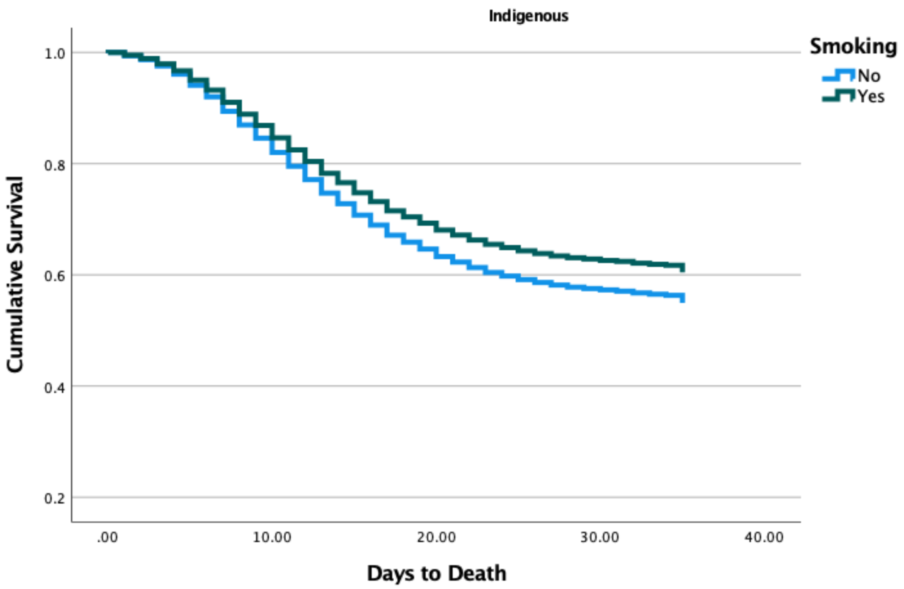
Indigenous
Smoking
No
Yes
Cumulative Survival
1.0
0.8
0.6
0.4
0.2
.00
10.00
20.00
30.00
40.00
Days to Death

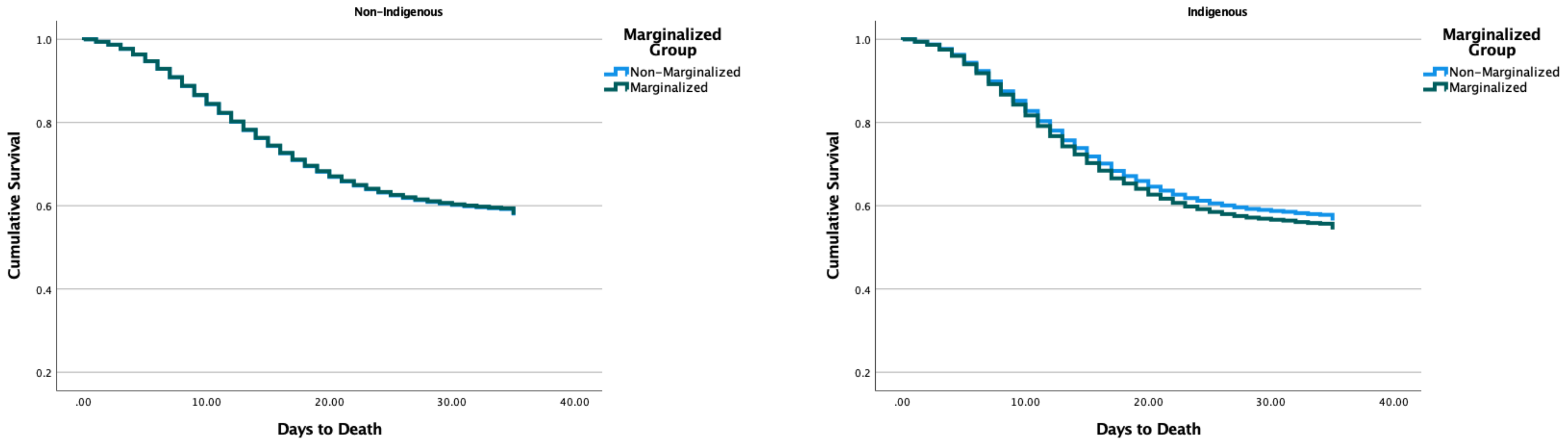
Non-Indigenous
Indigenous
Cumulative Survival
Days to Death
Marginalized Group
Non-Marginalized
Marginalized
1.0
0.8
0.6
0.4
0.2
.00
10.00
20.00
30.00
40.00

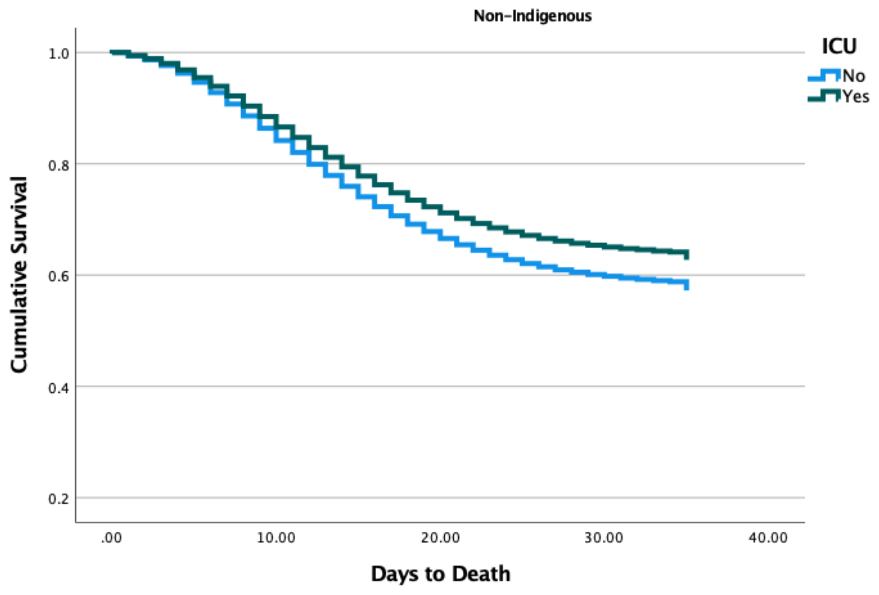
Non-Indigenous
ICU
No
Yes
Cumulative Survival
1.0
0.8
0.6
0.4
0.2
.00
10.00
20.00
30.00
40.00
Days to Death

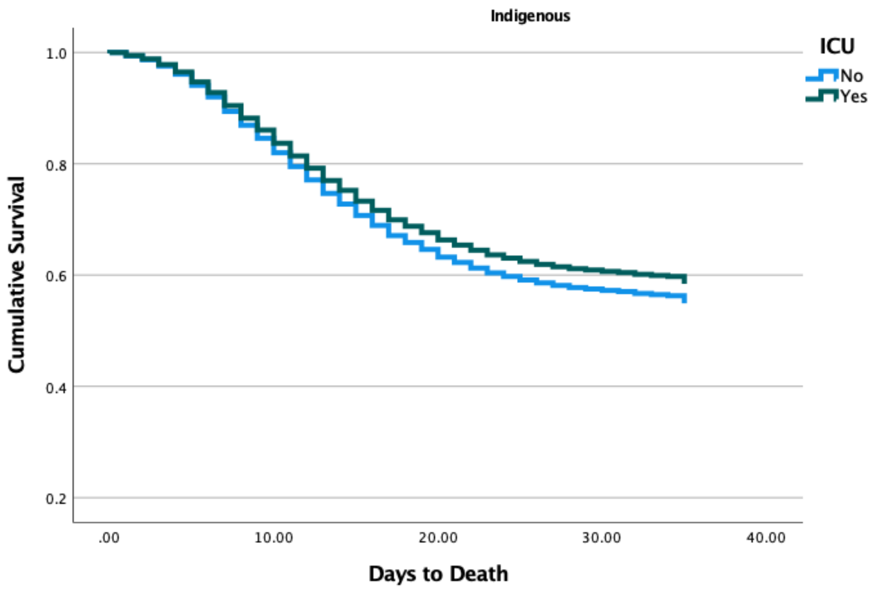
Indigenous
ICU
No
Yes
Cumulative Survival
1.0
0.8
0.6
0.4
0.2
.00
10.00
20.00
30.00
40.00
Days to Death

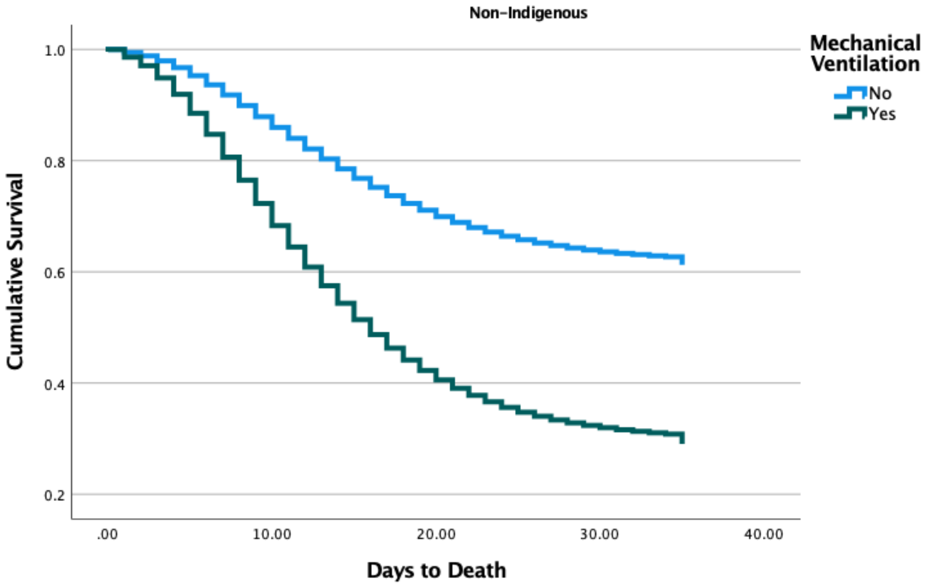
Non-Indigenous
Mechanical Ventilation
No
Yes
Cumulative Survival
1.0
0.8
0.6
0.4
0.2
.00
10.00
20.00
30.00
40.00
Days to Death

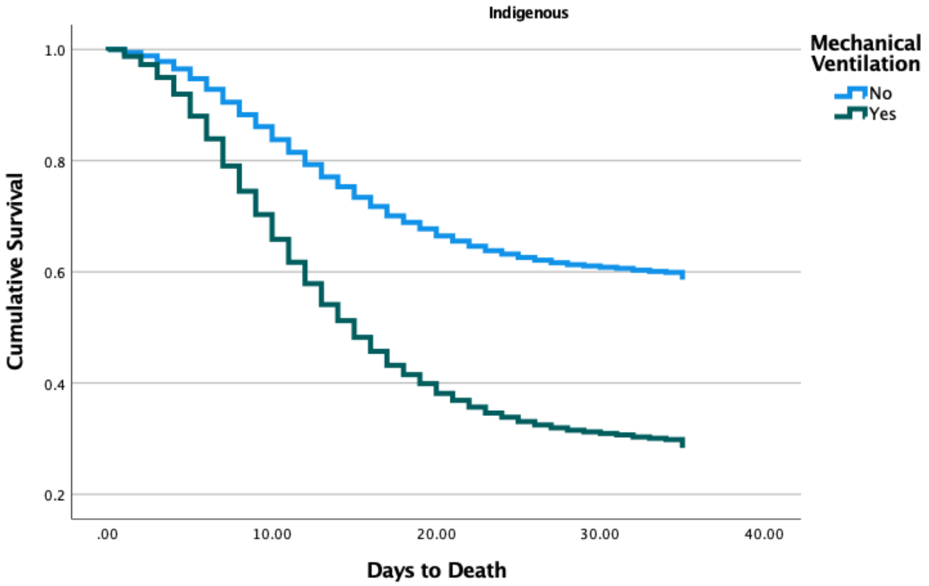
Indigenous
Mechanical Ventilation
No
Yes
Cumulative Survival
1.0
0.8
0.6
0.4
0.2
.00
10.00
20.00
30.00
40.00
Days to Death
